# Fracture healing: from molecular and cellular mechanisms to therapeutic strategies

**DOI:** 10.1038/s41392-026-02967-z

**Published:** 2026-09-22

**Authors:** Flurina Staubli, Renske Maria Tariro ten Ham, Jan Eelco Bergsma, Debby Gawlitta, Kenny Man

**Affiliations:** 1 Department of Oral and Maxillofacial Surgery & Special Dental Care, University Medical Center Utrecht, Utrecht, the Netherlands; 2 Regenerative Medicine Center Utrecht, Utrecht, the Netherlands; 3 Department of Epidemiology & Health Economics, Julius Center for Health Sciences and Primary Care, University Medical Center Utrecht, Utrecht, the Netherlands

**Keywords:** Translational research, Drug regulation

## Abstract

Fracture healing is a highly orchestrated regenerative process that depends on precise temporal and spatial coordination of immune, vascular, neural, and skeletal signaling programs. Disruption of these interconnected pathways, whether due to local injury factors, systemic disease, aging, or metabolic dysregulation, can impair repair and result in delayed union or nonunion. Understanding the molecular mechanisms that integrate inflammation resolution, progenitor cell fate decisions, neurovascular coupling, and endochondral bone formation is therefore essential for improving clinical outcomes. Recent advances show that fracture repair is governed by dynamic signaling networks linking immune cells, mesenchymal progenitors, endothelial and neural components, and the extracellular matrix. Key pathways regulating cytokine signaling, angiogenesis, mechanotransduction, hypoxia sensing, and osteochondral differentiation have emerged as critical determinants of healing efficiency and quality. These insights have reshaped therapeutic strategies, enabling the development of targeted interventions, including growth factor and anabolic therapies, immunomodulatory agents, cell– and gene-based approaches, extracellular vesicles, and biomaterial-assisted delivery systems. In this review, we synthesize current mechanistic understanding of both physiological and impaired fracture healing, with an emphasis on intercellular signaling and pathway-level regulation. We critically evaluate emerging targeted regenerative therapies in terms of their biological rationale, precision, and translational limitations, and discuss key barriers to clinical implementation. By shifting focus from descriptive pathology to actionable signaling frameworks, this review aims to guide the rational design of next-generation therapies for bone regeneration.

## Introduction

Bone fractures are a major and growing global health challenge, with more than 178 million incident cases and 455 million prevalent cases reported in 2019.^1^ Although most fractures heal with modern care, approximately 5–10% of fractures, particularly long-bone fractures, show delayed healing or progress to nonunion, imposing substantial socioeconomic costs through repeat surgery, prolonged rehabilitation, and lost productivity.^2,3^ This burden may be amplified in low-resource settings, where constraints in trauma care, infection control, and rehabilitation can worsen outcomes.^4,5^

This burden is expected to increase further because of population aging, the rising prevalence of osteoporosis and metabolic disease, and changing injury patterns.^1,6^ Fragility fractures predominate in older adults, whereas younger individuals in many low-resource settings more often sustain fractures related to traffic or occupational trauma.^4,5^ Systemic factors, including diabetes, chronic kidney disease, obesity, smoking, and sex hormone deficiency, further compromise repair. Together, these factors underscore that impaired fracture healing is a multifactorial clinical and biological problem.^1,7^

Fracture healing is a biologically and mechanically regulated regenerative process that proceeds through overlapping phases of inflammation, progenitor recruitment, angiogenesis, chondrogenesis, osteogenesis, and callus remodeling into lamellar bone.^2,8^ Disruption of these coordinated events by persistent inflammation, vascular insufficiency, metabolic dysfunction, infection, or mechanical instability can shift repair toward delayed union or nonunion.^9,10^

The canonical staged framework of fracture repair remains foundational. However, contemporary research increasingly shows that healing is regulated by broader, integrated networks involving signaling crosstalk, immune-skeletal and neuro-skeletal interactions, vascular specialization, mechanobiological control, and systemic pathological context.^9,11^ Recent advances include growing recognition of immune and neural regulation of repair, cell-free regenerative strategies such as extracellular vesicle (EV)-based therapies, and efforts to develop biomarker-guided and patient-stratified approaches to fracture care. Therapeutic development has also expanded beyond grafting and fixation adjuncts. Current and emerging strategies include growth factors, osteoanabolic agents, cell- and gene-based therapies, EV-based approaches, immunoregenerative and microbiome-targeted approaches, biomaterial-assisted delivery systems, and advanced surgical techniques. Despite this progress, clinical translation remains constrained by variable efficacy, inadequate patient stratification, manufacturing and quality-control challenges, safety and regulatory complexity, and uncertainty surrounding reimbursement and health technology assessment.^9^

Building on the staged framework of fracture repair established by Einhorn and Gerstenfeld,^2^ this review synthesizes current understanding of physiological and impaired fracture healing, with emphasis on the molecular, cellular, systemic, and translational factors that regulate repair. It also examines fracture healing in pathological and comorbid settings, evaluates established and emerging therapeutic strategies, and considers the biomarker, regulatory, and implementation barriers that continue to shape clinical application.

## Cellular players and phases of fracture healing

Fracture repair involves coordinated immune, stromal, skeletal, vascular, and neural cell populations that restore skeletal continuity and mechanical function. Classically, it progresses through inflammation, callus formation, and remodeling.^8^ This framework has been refined by evidence for immune–stromal crosstalk, vascular specialization, neural inputs, and post-transcriptional regulation, including microRNAs (miRNAs).^11,12^

### Inflammatory phase

Fracture-induced vascular disruption and hematoma formation create a provisional matrix that initiates repair. Platelets rapidly aggregate to achieve hemostasis and release growth factors and cytokines that recruit inflammatory cells and mesenchymal stromal/stem cells (MSCs) to the injury site.^13,14^ Neutrophils infiltrate within hours and, beyond their roles in debris clearance and host defense, participate in early inflammatory signaling that can influence subsequent repair.^15–18^ Monocytes subsequently enter the fracture hematoma and differentiate into macrophages with dynamic activation states. Early inflammatory macrophages support tissue clearance and antimicrobial defense, whereas later reparative macrophages promote inflammation resolution, angiogenesis, and progenitor differentiation. Although the M1/M2 framework is simplified, it remains a useful shorthand for this functional transition.^19,20^ Other immune and stromal populations further shape the early fracture environment. Regulatory T cells help restrain excessive inflammation and support reparative immune states, while mast cells may further influence vascular permeability, leukocyte recruitment, and local immune activation.^21–23^ As the hematoma organizes into granulation tissue and an early fibrovascular callus, mesenchymal progenitors and stromal cells contribute to matrix deposition, defect stabilization, and angiogenic invasion.^24^ Emerging evidence suggests that marrow adipocytes and their secreted factors can influence skeletal progenitor fate.^25,26^ In parallel, immune and stromal cells release EVs and miRNAs that modulate local progenitor responses and may influence the reparative microenvironment.^12,17^ Collectively, these processes convert the fracture hematoma into fibrovascular repair tissue that supports vascular ingrowth and subsequent bone formation.

### Repair phase

As inflammation resolves, the repair phase proceeds through soft- and hard callus formation and depends on the recruitment, proliferation, and differentiation of progenitor cells. Periosteal progenitors are a major source of callus chondrocytes and osteoblasts during fracture repair, whereas bone marrow–derived progenitors likely provide paracrine support and serve as a secondary reservoir.^27^ Local environmental cues determine lineage allocation within the callus. In well-oxygenated, mechanically stable regions, MSCs undergo osteogenic differentiation and form intramembranous bone at cortical surfaces. In contrast, under hypoxic or mechanically stressed conditions, they adopt a chondrogenic program and generate the cartilaginous soft callus that stabilizes the fracture.^11^ Chondrocytes then mature and undergo hypertrophy, promoting matrix mineralization and replacement by woven bone.^11,28,29^ Lineage-tracing studies indicate that some hypertrophic chondrocytes can directly contribute to the osteoblast pool, revising the traditional view that they are terminally differentiated.^30–32^ Angiogenesis is essential for progression from soft to hard callus. New capillaries sprout from adjacent vessels and establish networks that deliver oxygen and nutrients required for osteoblast activity and matrix mineralization.^11,33,34^ Specialized endothelial populations have also been described in experimental skeletal and preclinical fracture studies. Type H vessels are associated with osteoprogenitor-rich regions and angiogenic–osteogenic coupling, whereas type L vessels more closely resemble sinusoidal marrow networks.^35,36^ Although these subtypes have been defined mainly in experimental systems, they have refined understanding of how vascular identity might influence callus maturation. Immune cells continue to shape the reparative environment. Reparative macrophages predominate and support progenitor recruitment, chondrogenesis, osteogenesis, and ongoing resolution of inflammation, while regulatory T cells help maintain a pro-regenerative milieu.^19,37^ Dendritic and B cells may further modulate adaptive immune responses and osteoclast-related remodeling.^37^ Reinnervating sensory nerve fibers may release neuropeptides, while sympathetic fibers may release catecholamines that influence angiogenesis, osteoblast activity, and local immune tone, adding a neuro-skeletal layer of regulation.^38^ miRNAs provide an additional post-transcriptional mechanism controlling chondrogenic and osteogenic differentiation programs.^12^ Clearance of residual inflammatory cells and matrix debris accompanies the transition to hard callus formation, culminating in deposition of mineralized woven bone.^10^

### Remodeling phase

The remodeling phase is the final and longest stage of fracture healing, during which temporary woven bone is progressively replaced by mature lamellar bone.^2^ This phase reshapes the callus through tightly coupled cycles of resorption and formation, restoring bone architecture according to mechanical demand. Osteoclasts, large multinucleated cells of hematopoietic origin, attach to bone surfaces and degrade mineralized and organic matrix within resorption lacunae.^39^ Reversal cells then prepare these surfaces for new bone deposition. Osteoblasts repopulate resorbed sites, secrete osteoid, and mineralize it to form lamellar bone, restoring bone geometry and mechanical strength.^40–42^ Within the matrix, osteocytes act as mechanosensors, detecting strain and microdamage through their canalicular network and regulating osteoclast and osteoblast activity to align remodeling with local loading conditions.^43^ Bone-lining cells, quiescent osteoblast derivatives, cover inactive bone surfaces, contribute to coupling between resorption and formation, and help initiate subsequent bone formation.^44^ Although acute inflammatory activity largely subsides, adaptive immune cells may persist and continue to modulate osteoclast and osteoblast activity.^45^ Through the coordinated actions of skeletal, stromal, vascular, neural, and immune cells, remodeling completes the conversion of the fracture callus into organized lamellar bone and restores structural and mechanical integrity.

Taken together, these phases define fracture healing as a regulated regenerative process that depends on coordinated transitions between inflammation, fibrovascular organization, callus formation, vascularization, ossification, and remodeling. Disruption of these transitions underlies delayed healing and nonunion and provides the biological rationale for the signaling pathways and therapeutic strategies discussed in subsequent sections.

## Signaling networks and regulatory axes in fracture healing

Fracture repair is controlled by signaling networks that integrate inflammatory, skeletal, vascular, mechanical, metabolic, and neural inputs across successive healing phases. These pathways help coordinate inflammatory activation, progenitor recruitment, lineage specification, osteoangiogenic coupling, mechanoadaptation, and remodeling of cartilage and woven bone into lamellar bone.^2,11,46^ The discussion below emphasizes fracture-specific evidence while incorporating broader skeletal biology where direct fracture data remain limited.

### Intracellular signaling integrators

Intracellular signaling hubs translate growth factors, cytokines, mechanical stimuli, neuroendocrine signals, and metabolic cues into cell-type- and stage-specific responses. By regulating survival, proliferation, migration, differentiation, and metabolic adaptation, they influence inflammation resolution, progenitor recruitment, angiogenesis, callus maturation, and remodeling. Their output depends on timing, intensity, and tissue context; signals that support repair early may become maladaptive if prolonged or excessive.^11,46–48^

#### PI3K–Akt–mTOR

**Fig. 1:**
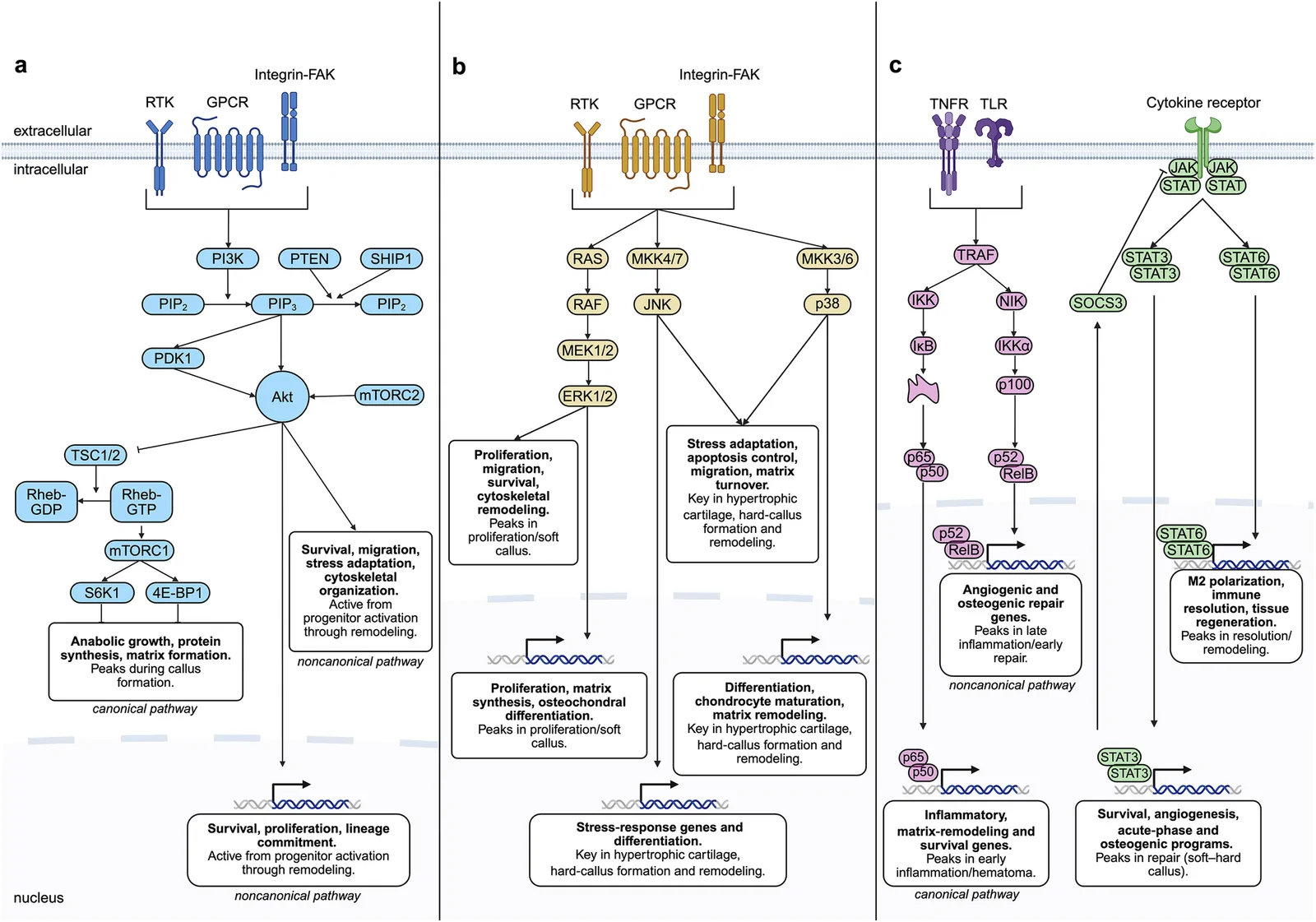
Intracellular signaling hubs in fracture healing. Major intracellular integrators coordinate cell survival, proliferation, differentiation, migration, and metabolism across fracture repair. **a** PI3K–Akt–mTOR pathway: RTK, GPCR, and integrin–FAK inputs activate PI3K, generating PIP₃, which recruits Akt for activation by PDK1 and mTORC2. Akt-dependent mTORC1 signaling promotes anabolic metabolism and matrix production, whereas additional Akt-dependent branches support survival, migration, stress adaptation, and cytoskeletal organization. PTEN converts PI(3,4,5)P₃ to PI(4,5)P₂, whereas SHIP1 converts PI(3,4,5)P₃ to PI(3,4)P₂, thereby negatively regulating PI3K–Akt signaling. **b** MAPK cascades: ERK1/2, JNK, and p38 branches regulate proliferation, migration, survival, stress responses, matrix turnover, and differentiation in a context-dependent manner. **c** NF-κB and JAK/STAT axis: TLRs and receptors such as TNFR1 activate canonical NF-κB signaling, typically through IκB degradation and p65/p50 nuclear translocation, whereas selected TNF-receptor-superfamily members activate noncanonical NF-κB signaling through NIK- and IKKα-dependent p100 processing and p52/RelB-dependent programs. Cytokine receptors activate JAK/STAT signaling, including STAT3- and STAT6-associated programs, with SOCS3-mediated feedback constraining cytokine signaling. Pathways are active across multiple repair phases; any phase placement in the figure indicates predominant or illustrative activity rather than strict phase exclusivity. Created with BioRender.com

The phosphatidylinositol 3-kinase–Akt–mechanistic target of rapamycin (PI3K–Akt–mTOR) pathway (Fig. 1a) links extracellular cues to cell survival, anabolic metabolism, and growth-related responses during skeletal repair. In fracture healing, available evidence supports a role for this pathway in progenitor expansion, matrix production, angiogenic support, and progression toward mineralized tissue formation, although some mechanistic details are derived from broader skeletal biology rather than fracture-specific models. The pathway is activated downstream of receptor tyrosine kinases (RTKs), G-protein-coupled receptors (GPCRs), and integrin–focal adhesion kinase (FAK) complexes, which stimulate PI3K to generate phosphatidylinositol-3,4,5-trisphosphate (PIP₃) from phosphatidylinositol-4,5-bisphosphate (PIP₂).^49,50^ Lipid phosphatases, including phosphatase and tensin homolog (PTEN) and Src homology-2-containing inositol phosphatase 1 (SHIP1), oppose this step by dephosphorylating PIP₃. In osteoblast-lineage cells, PTEN loss increases Akt signaling and osteogenic activity, supporting a role for PI3K–Akt signaling in osteoblast anabolic function.^51,52^ By contrast, SHIP1 has been implicated in the regulation of MSC osteolineage commitment and osteoclast-related responses, although its role in fracture repair remains less clearly defined.^53^ PIP₃ recruits Akt to the plasma membrane, where it is activated by phosphoinositide-dependent kinase-1 (PDK1) and mTOR complex 2 (mTORC2). Akt then signals through canonical and noncanonical branches. Through mTOR complex 1 (mTORC1), it promotes anabolic metabolism, protein synthesis, matrix production, and growth, whereas other Akt-dependent branches contribute to survival, oxidative-stress resistance, vascular responses, and cytoskeletal organization. Through these combined actions, PI3K–Akt–mTOR signaling may influence angiogenesis, migration, and tissue remodeling during fracture repair.^49^ Dynamic regulation of this pathway appears important: activation can support osteogenesis and repair-related anabolic responses, whereas persistent or dysregulated signaling may alter lineage allocation, autophagy, or callus maturation.^50^ PI3K–Akt–mTOR therefore functions less as a simple pro-healing switch than as a context-dependent regulator of anabolic and remodeling responses.

#### MAPK

The mitogen-activated protein kinase (MAPK) network comprises three major cascades (Fig. 1b): extracellular signal-regulated kinases (ERKs), c-Jun N-terminal kinases (JNKs), and p38 kinases. Among these, ERK1/2 is particularly well characterized in osteoblast biology and skeletal repair, where it links growth-factor and mechanical inputs to progenitor proliferation, matrix production, and anabolic responses that are relevant to callus formation. RTKs, GPCRs, and integrin–FAK complexes activate the small GTPase RAS, which drives the RAF–MAPK kinase 1/2 (MEK1/2)–ERK1/2 cascade.^54,55^ Activated ERK1/2 regulates survival, cytoskeletal remodeling, matrix turnover, and transcriptional programs that support proliferation, extracellular matrix (ECM) production, and differentiation. Importantly, ERK signaling is not uniformly beneficial: transient activation promotes progenitor proliferation and lineage commitment, whereas sustained activation in mature osteoblasts can restrain terminal differentiation.^54,56^ The p38 and JNK branches are also implicated in stress-responsive regulation of osteoblasts and skeletal progenitors, but the evidence cited here is broader skeletal biology rather than direct fracture-callus evidence. Accordingly, p38 and JNK signaling should be viewed as context-dependent modulators of osteogenic differentiation, survival, and apoptosis under hypoxic, inflammatory, or mechanical stress conditions.^54,55^ This context dependence is particularly relevant in disorders marked by RAS–MAPK hyperactivation. Neurofibromatosis type 1, a RASopathy, is associated with skeletal abnormalities such as tibial pseudarthrosis,^57^ and preclinical models demonstrate that excessive ERK signaling impairs osteogenic differentiation but can be partially rescued by MEK inhibition.^58^ These observations underscore that MAPK signaling must be tightly regulated. Although pathological ERK hyperactivation can disrupt bone formation, appropriately regulated MAPK activity is likely to support normal skeletal repair, with pathway output depending on cell type, timing, and inflammatory context.

#### NF-κB and JAK/STAT

Cytokine-activated transcriptional systems, particularly nuclear factor-κB (NF-κB) and Janus kinase/signal transducer and activator of transcription (JAK/STAT) signaling (Fig. 1c), form a major inflammatory axis linking immune-derived cues to fracture repair. At the fracture site, they help determine whether the inflammatory hematoma transitions into a reparative environment or remains locked in persistent inflammatory activation. NF-κB is activated downstream of TNF-family receptors and TLRs through adaptor complexes that include tumor necrosis factor receptor-associated factors (TRAFs). These in turn can activate the IκB kinase (IKK) complex. IKK-mediated phosphorylation of IκB leads to its degradation and release of NF-κB dimers, typically p65/p50 in canonical signaling. These dimers then translocate to the nucleus and induce genes involved in inflammation, matrix remodeling, and survival.^59–61^ Noncanonical NF-κB signaling, mediated by NF-κB-inducing kinase (NIK)- and IκB kinase α (IKKα)-dependent p100 processing and p52/RelB dimers, can regulate immune, angiogenic, osteoclast-related, and osteogenic programs depending on receptor context and cell type.^61–63^ In fracture healing, early NF-κB activation is thought to support debris clearance, innate defense, and recruitment of reparative cells. However, excessive or persistent activation can prolong inflammation, impair osteogenic differentiation, and predispose to delayed healing or nonunion.^63^ JAK/STAT signaling also contributes to both inflammatory and reparative phases. Cytokine-bound receptors activate JAK kinases, which phosphorylate STAT factors, particularly STAT3 and STAT6. These factors then dimerize, translocate to the nucleus, and regulate genes involved in macrophage polarization, angiogenesis, survival, and tissue adaptation.^60^ Broader inflammatory-signaling literature indicates that STAT3 and NF-κB can interact bidirectionally, with cytokine-driven NF-κB activity enhancing STAT signaling, and STAT3, in some contexts, limiting or reshaping NF-κB-driven inflammatory programs. Suppressor of cytokine signaling (SOCS) proteins, especially SOCS3, add a further layer of control by constraining cytokine signaling and helping restore signaling balance.^64^ Together, the NF-κB–JAK/STAT axis helps determine whether early inflammation resolves into a pro-regenerative milieu or persists in a way that disrupts callus progression. This may help explain why infection, aging, metabolic disease, and systemic inflammatory comorbidities alter healing trajectories. Therapeutic suppression of persistent cytokine signaling may be beneficial, but it risks weakening early immune programs required for debris clearance, antimicrobial defense, and progenitor recruitment.

### Early signaling cascades: initiation, recruitment & regulation

Fracture healing begins with vascular rupture and hematoma formation, creating a hypoxic, damage-associated molecular pattern (DAMP)-rich environment. DAMPs can activate Toll-like receptors 2 and 4 (TLR2/4) on innate immune and stromal cells, while intracellular danger sensing can engage the NOD-like receptor pyrin domain-containing 3 (NLRP3) inflammasome. Together, these pathways can promote interleukin-1β (IL-1β) maturation, leukocyte recruitment, vascular activation, and progenitor mobilization.^11,65^

#### TNF/TNFR axis

**Fig. 2:**
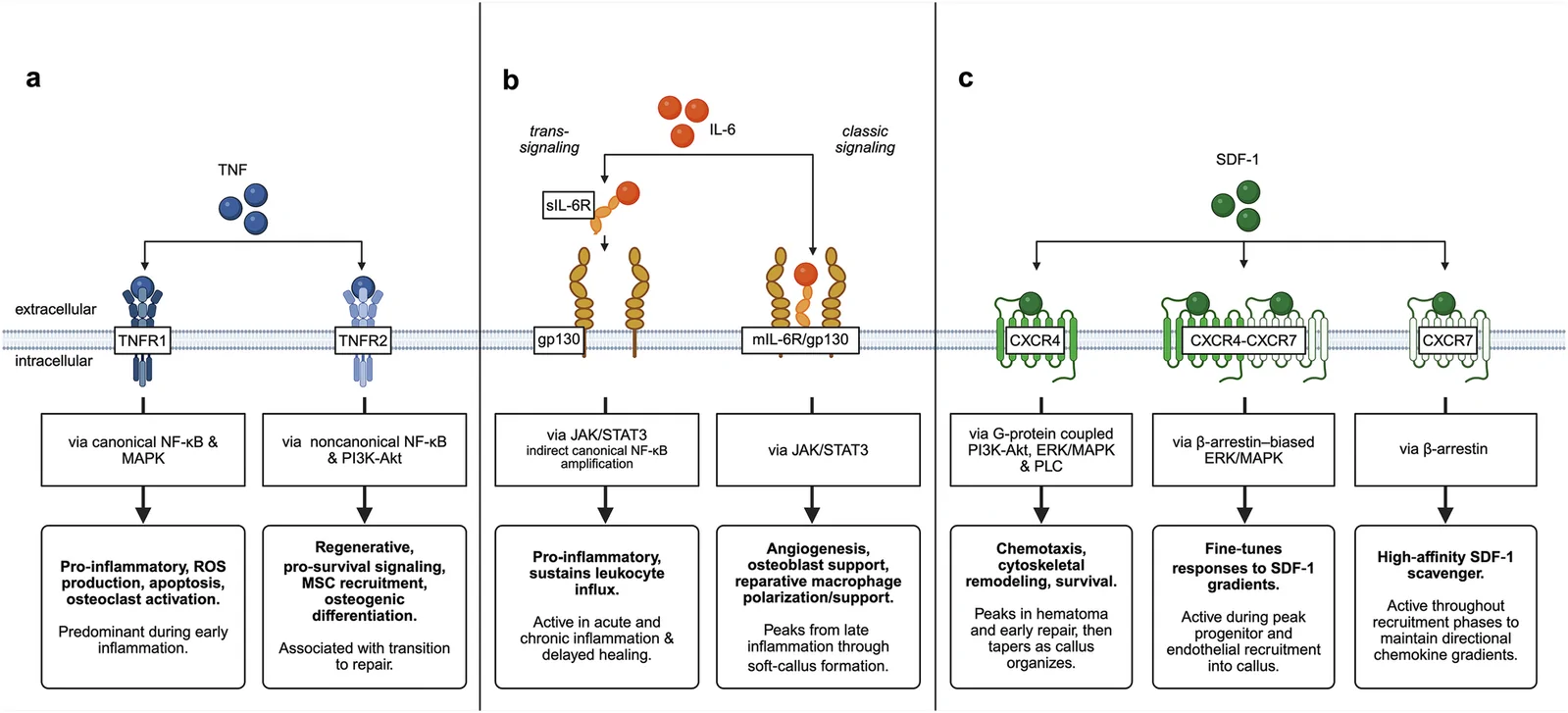
Key Cytokine and Chemokine Signaling Switches in Early Fracture Healing. This figure highlights signaling axes with context-dependent receptor or pathway switches that govern the inflammation-to-repair transition: **a** TNFR1-biased early inflammatory signaling and TNFR2-associated reparative signaling, rather than a strict TNFR1-to-TNFR2 switch; **b** IL-6 signaling duality, including trans- versus classic signaling; and **c** SDF-1 receptor interplay involving CXCR4 and CXCR7 gradient refinement. Pathway activity overlaps across repair phases; phase labels indicate predominant or illustrative activity rather than exclusive timing. Other key cytokines including IL-1β (IL-1R → NF-κB/MAPK; pro-inflammatory), IL-17A (IL-17RA → NF-κB/MAPK; pro-inflammatory and osteogenic priming in early repair), IL-4 (IL-4R → JAK/STAT6; anti-inflammatory and reparative macrophage polarization), and IL-10 (IL-10R → JAK/STAT3; anti-inflammatory and osteoblast support) act through constitutive receptor-pathway pairs and are described in the text. Created with BioRender.com

Immediately after fracture, tumor necrosis factor alpha (TNF) released by macrophages, neutrophils, and endothelial cells (ECs) contributes to early inflammatory activation and can signal through TNF receptor 1 (TNFR1), engaging canonical NF-κB and MAPK cascades. In ECs, TNFR1 signaling induces intercellular adhesion molecule 1 (ICAM-1) and vascular cell adhesion molecule 1 (VCAM-1), promoting leukocyte adhesion and transmigration.^66^ In MSCs and other stromal or immune-responsive cells, TNF signaling can induce pro-inflammatory mediators, including IL-6, matrix metalloproteinases (MMPs), and chemokines, thereby contributing to inflammatory amplification, ECM turnover, and cell-recruitment programs. TNF-related signaling may also influence MSC recruitment pathways, including stromal cell-derived factor-1 (SDF-1)/C-X-C chemokine receptor type 4 (CXCR4)-mediated chemotaxis, although this should be interpreted as a broader stromal-cell mechanism rather than definitive fracture-specific evidence.^67^ In myeloid cells, TNF/TNFR signaling contributes to inflammatory activation and may support antimicrobial and debris-clearance functions, while TNFR1 signaling can also promote apoptosis of short-lived inflammatory cells such as neutrophils.^68^ As repair progresses, TNFR2-associated signaling has been proposed to contribute to reparative responses, interacting with noncanonical NF-κB, PI3K–Akt, and ERK/MAPK pathways in bone-related cells. Crosstalk among these cascades may promote angiogenesis in ECs, osteogenic priming in MSCs, and reparative macrophage polarization. Thus, the transition from early TNFR1-dominant inflammatory signaling toward greater TNFR2-associated repair signaling is best interpreted as a proposed regulatory shift rather than a fully established temporal switch in human fracture healing (Fig. 2a).^67,69^ Successful healing appears to depend less on TNF abundance alone than on appropriate timing, receptor context, and cellular target. Accordingly, broad TNF suppression may be counterproductive if it disrupts angiogenesis, progenitor recruitment, or progression into repair.

#### Interleukin networks

Interleukin signaling, like TNF signaling, shifts from pro-inflammatory to pro-regenerative phases during fracture healing.^47^ In the acute phase, IL-1β, induced by DAMP-triggered TLR activation and NLRP3 inflammasome signaling, is further upregulated by inflammatory and hypoxic cues. It activates canonical NF-κB and MAPK pathways in macrophages, stromal fibroblasts, and ECs, promoting secretion of pro-inflammatory cytokines, chemotactic factors, and MMPs. These responses support leukocyte recruitment, progenitor mobilization, and ECM remodeling.^70,71^ IL-6, induced downstream of IL-1β and TNF, exerts biphasic effects. Trans-signaling via soluble IL-6R (sIL-6R) engages gp130 on cells lacking membrane IL-6R and activates predominantly JAK/STAT3, with parallel ERK/MAPK and PI3K–Akt signaling. In broader inflammatory contexts, this mode is associated with leukocyte recruitment and inflammatory amplification. By contrast, classic signaling via membrane IL-6R (mIL-6R)/gp130 is restricted to IL-6R-positive cells and supports STAT3-linked programs that promote ECM synthesis, vascular endothelial growth factor (VEGF) production, angiogenesis, and progression through soft callus formation. This signaling split allows IL-6 to amplify early inflammation while later supporting resolution and repair (Fig. 2b).^72^ IL-17A, produced by γδT cells and T helper 17 (Th17) lymphocytes, has been linked in preclinical studies to enhanced bone regeneration, connecting inflammatory T-cell responses to osteogenic repair programs.^73^ During later repair, anti-inflammatory cytokines, including IL-10 and IL-4, become more prominent. These cytokines are generally associated with inflammation resolution and reparative immune polarization, helping to limit persistent NF-κB-driven inflammatory signaling and support progression toward tissue repair.^47,74,75^ Interleukin networks therefore contribute to regulation of the transition from fracture hematoma organization to callus formation through a controlled shift between inflammatory and reparative signaling states. IL-6 trans- versus classic signaling illustrates how signaling mode and timing shape outcome, but these modes may overlap rather than acting as mutually exclusive phases.

#### SDF-1–CXCR4 axis

The SDF-1/CXCR4/CXCR7 chemokine system links early inflammatory and hypoxic signaling to progenitor recruitment during fracture repair (Fig. 2c).^11,76^ Early post-fracture hypoxia is thought to support SDF-1 expression in stromal and vascular cell populations, although some evidence for hypoxia-induced SDF-1 derives from non-fracture stromal-cell systems.^77^ SDF-1–CXCR4 signaling activates PI3K–Akt, ERK/MAPK, and phospholipase C (PLC) pathways, regulating cytoskeletal remodeling and calcium flux to guide MSCs, endothelial progenitors, and hematopoietic cells toward the fracture site. CXCR7 can modulate SDF-1 availability by acting as a high-affinity scavenger receptor that internalizes and degrades SDF-1, thereby helping refine chemokine gradients and spatially focus recruitment. In cells expressing both receptors, CXCR4–CXCR7 interplay may further modulate migration, retention, and proliferation during repair.^76^ CXCR7 can also signal through β-arrestin-mediated ERK activation, although this mechanistic evidence is derived largely from broader chemokine biology rather than fracture-specific models.^78^ As vascularization improves oxygenation, SDF-1 gradients are expected to decline, helping shift the fracture environment from cell recruitment toward matrix maturation and vascular integration.^76^ The translational potential of this axis lies in promoting endogenous progenitor homing and retention, but therapeutic benefit will likely depend on recreating appropriate spatial and temporal chemokine gradients rather than simply increasing SDF-1 exposure.

#### Neural and neurovascular signaling

Alongside cytokine and chemokine cues, neural inputs are increasingly recognized as modulators of fracture repair. Sensory nerve fibers near sites of skeletal injury can release neuropeptides, including substance P (SP) and calcitonin gene-related peptide (CGRP), which have been linked to vascular, stromal, osteogenic, immune, and osteoclast-related responses.^79,80^ Through their respective receptor systems, these neuropeptides may influence angiogenesis, progenitor activity, inflammatory tone, and remodeling, although much of the mechanistic evidence derives from broader bone-regeneration or non-fracture models. Sympathetic signaling is also implicated in fracture repair; for example, sympathetic activation has been linked to altered bone marrow myelopoiesis and enhanced fracture healing in preclinical work, supporting a connection between autonomic tone, immune regulation, and repair.^81^ Nerve growth factor (NGF) is among the neurotrophic signals implicated in fracture repair, where sensory reinnervation and neurotrophin signaling may influence vascularization, inflammatory tone, and osteogenic activity within the regenerating callus.^38^ Together, these pathways suggest that altered innervation can influence vascularization, inflammatory tone, and osteogenic activity within the fracture callus. However, evidence for neural regulation in fracture healing remains largely preclinical, and the relative contributions of reparative, compensatory, and maladaptive neural inputs in human fractures remain incompletely defined. Neural signaling is therefore best viewed as an emerging modulatory axis rather than a clinically validated therapeutic target.

### Morphogen pathways governing lineage specification

During and following the inflammatory phase, morphogen pathways help guide progenitor cells toward osteogenic and chondrogenic fates. Within defined temporal and spatial windows, these pathways coordinate differentiation, ECM synthesis, and tissue patterning in the developing callus. In doing so, they can influence how the fracture environment is partitioned into zones of intramembranous and endochondral bone formation and whether repair progresses in an orderly manner or shifts toward impaired healing.^82^

#### TGF-β superfamily

**Fig. 3:**
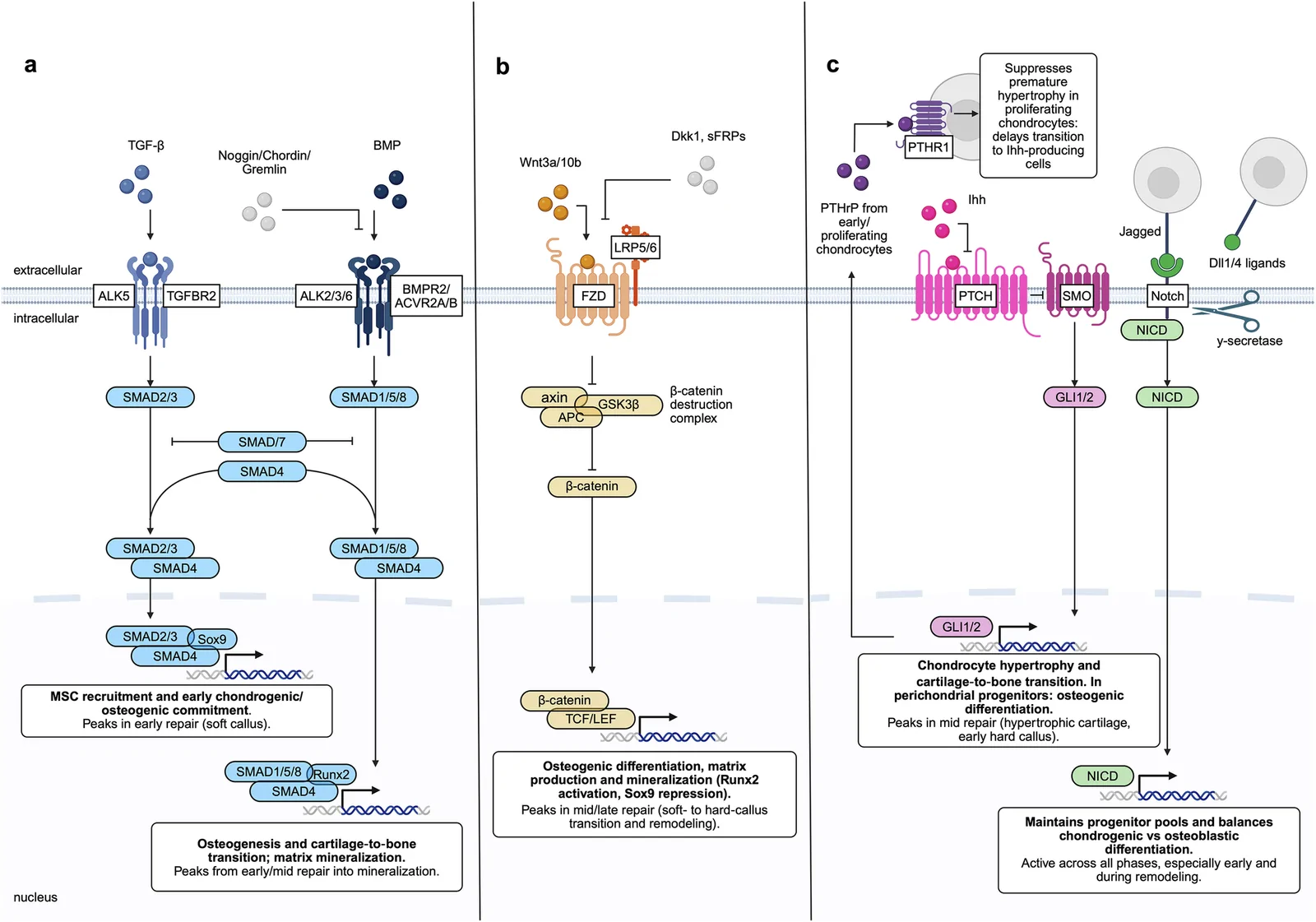
Morphogen pathways governing MSC lineage specification. This figure illustrates three major morphogenetic signaling pathways that guide MSC differentiation during fracture healing. **a** TGF-β Superfamily: TGF-β and BMP ligands activate Smad-dependent pathways (SMAD1/5/8 for BMP; SMAD2/3 for TGF-β), which complex with SMAD4 and cooperate with lineage-specific transcription factors (Runx2 for osteogenesis; Sox9 for chondrogenesis) to drive MSC recruitment and lineage commitment. Inhibitory SMADs provide intracellular negative feedback, while secreted antagonists Noggin, Chordin, and Gremlin act primarily as BMP antagonists. **b** Canonical Wnt/β-Catenin: Wnt ligands stabilize β-catenin, which complexes with TCF/LEF to activate osteogenic genes and can suppress Sox9-associated chondrogenic programs in a timing-dependent manner, promoting osteoblast differentiation. **c** Hedgehog and Notch: Ihh drives chondrocyte hypertrophy and osteogenic differentiation in perichondrial progenitors; PTHrP produced by early/proliferating chondrocyte or perichondrial/periarticular regions signals through PTH1R to suppress premature hypertrophy and regulate the pace of endochondral ossification. Notch signaling, activated by Jagged and Delta-like ligands such as Dll1 and Dll4, maintains progenitor pools and balances chondrogenic versus osteoblastic differentiation at periosteal fronts. Phase labels indicate predominant activity and do not imply that these pathways are absent from other repair stages. Noncanonical pathways and effects on endothelial and immune cells are described in the text (Section “TGF-β Superfamily”). Created with BioRender.com

Transforming growth factor beta (TGF-β) and bone morphogenetic proteins (BMPs) are central regulators of lineage specification and matrix production during fracture repair (Fig. 3a).^83^ Ligands, including BMP-2/4/7 and TGF-β1/3, are released by multiple cell types within the repair environment, including platelets, osteoblast-lineage cells, chondrocytes, ECs, and damaged ECM components. They signal through type I and type II serine-threonine kinase receptors.^84^ BMPs and TGF-β activate receptor-regulated Smads: Smad1/5/8 for BMPs and Smad2/3 for TGF-β. These Smads complex with Smad4 and translocate to the nucleus, where they cooperate with transcription factors such as Runx2 and Sox9 to drive osteogenic and chondrogenic gene programs. Pathway output is further modulated by inhibitory Smads, and by secreted BMP antagonists such as noggin, chordin, and gremlin. miRNAs targeting receptors and transcriptional cofactors provide additional regulation.^83,85^ Within the fracture callus, TGF-β/BMP signaling is phase- and region-specific. BMP-2 is most strongly associated with early osteogenic and chondrogenic differentiation, whereas TGF-β1 has been linked more closely to progenitor recruitment, matrix synthesis during chondrogenesis, and endochondral ossification. However, TGF-β activity is highly context dependent: it can support recruitment and matrix production while also restraining osteoblast differentiation or angiogenesis depending on cell type and stage. In contrast, BMP signaling is generally more consistently associated with osteogenesis and can be coupled to osteoblast-derived VEGF signaling. During later callus maturation, BMP signaling supports osteoblast differentiation and mineralization.^86–88^ BMPs and their inhibitors also show region-specific localization across the callus rather than being confined to periosteal osteogenic fronts.^86^ Endogenous BMP-2 appears necessary for fracture initiation or the earliest steps of repair.^87^ Beyond MSC lineage commitment, canonical TGF-β/BMP signaling regulates transcriptional programs in differentiated osteoblasts, chondrocytes, ECs, and immune cells, influencing matrix synthesis, angiocrine factor production, and immune modulation. Noncanonical, Smad-independent pathways, including TAK1-mediated MAPK signaling, PI3K–Akt, and Rho GTPases, can also contribute to survival, migration, and VEGF-driven angiogenesis in osteogenic and endothelial populations.^83,88^ Together, the TGF-β/BMP axis contributes to lineage specification, tissue patterning, vascular coupling, and the tempo of callus maturation.

#### Wnt/β-Catenin signaling

The Wingless/Integrated (Wnt)–β-catenin network regulates osteogenic commitment, callus maturation, and remodeling during fracture repair (Fig. 3b).^89^ Canonical ligands such as Wnt3a and Wnt10b, described in bone-related cell populations including osteoblast-lineage, endothelial, and immune cells, bind Frizzled (FZD) and low-density lipoprotein receptor-related protein 5/6 (LRP5/6) receptors. This inhibits the β-catenin destruction complex and stabilizes cytosolic β-catenin. Stabilized β-catenin translocates to the nucleus, where it complexes with T-cell factor/lymphoid enhancer-binding factor (TCF/LEF) to activate osteogenic gene programs and can repress Sox9-associated chondrogenic programs in a timing-dependent manner, favoring osteoblast differentiation over chondrogenesis. Secreted inhibitors such as Dickkopf-1 (Dkk1) and secreted Frizzled-related proteins (sFRPs) further modulate canonical Wnt output.^90^ Canonical Wnt activity increases during the soft-to-hard callus transition and cooperates with BMP–Smad1/5/8 signaling to drive osteogenic maturation. By contrast, TGF-β–Smad2/3 signaling persists in cartilaginous regions to support chondrogenesis. Timing is critical: premature or excessive activation may suppress the cartilaginous intermediate required for normal endochondral repair, whereas insufficient later activation can impair osteoblast maturation and mineralization.^91^ In addition to regulating lineage commitment, canonical Wnt signaling influences multiple cell populations involved in repair. In osteoblasts, β-catenin upregulates osteoprotegerin (OPG) and suppresses receptor activator of nuclear factor κB ligand (RANKL), thereby coupling bone formation to reduced resorption. It may also interact with mechanotransduction pathways such as FAK–YAP/TAZ to support mineralization and structural organization of newly formed bone.^90,92^ Noncanonical ligands such as Wnt5a and Wnt11, signal through planar cell polarity and calcium-dependent pathways. Through Rho GTPases and FAK, they regulate cytoskeletal tension, polarity, and mechanosensitivity, and may help coordinate cellular orientation under mechanical load. In some contexts, noncanonical Wnt signaling has also been associated with osteoclastogenesis and resorptive activity.^93,94^ Canonical and noncanonical Wnt pathways therefore link lineage specification to the spatial and mechanical organization of the fracture callus. Therapeutic targeting will require careful control of timing and signaling balance.

#### Hedgehog and Notch pathways

Hedgehog and Notch signaling help fine-tune the cartilage-to-bone transition during fracture repair (Fig. 3c). Indian hedgehog (Ihh) is the dominant hedgehog ligand in endochondral fracture healing, whereas Sonic hedgehog may be re-expressed in some injury settings, although its role in fracture repair remains less clearly defined. Ihh acts on chondrocytes to promote hypertrophy and VEGF production and on adjacent perichondral progenitors to induce osteogenic differentiation. In canonical Hedgehog signaling, Ihh binding to Patched (PTCH) relieves PTCH-mediated inhibition of Smoothened (SMO) and activates GLI-dependent transcription; however, SMO-independent Hedgehog regulation of the cartilage-to-bone transition has also been reported.^95,96^ Ihh also stimulates parathyroid hormone-related protein (PTHrP) expression in early/proliferating chondrocytes, periarticular, or perichondrial regions. PTHrP, or systemic parathyroid hormone (PTH), then signals through parathyroid hormone 1 receptor (PTH1R) on proliferating chondrocytes to suppress premature hypertrophy and regulate the pace of ossification. This feedback architecture helps maintain controlled endochondral conversion.^97^ PTH also acts on osteoblasts, where it suppresses apoptosis and enhances differentiation, thereby promoting bone formation.^98^ Insulin-like growth factor-1 (IGF-1) further supports this transition by activating IGF-1 receptor (IGF-1R) on MSCs, chondrocytes, and osteoblasts. Through PI3K–Akt and MAPK signaling, IGF-1 possibly reinforces chondrogenesis during soft callus formation and supports osteoblast proliferation and matrix mineralization during hard callus formation.^99,100^ The Notch pathway is activated through direct cell–cell contact, in which Notch receptors bind Jagged (Jagged1/2) or Delta-like (Dll1/4) ligands expressed by neighboring cells, including periosteal progenitors and ECs. This triggers γ-secretase-mediated release of the Notch intracellular domain (NICD), which translocates to the nucleus and induces Hes and Hey transcriptional repressors. In MSCs and skeletal progenitors, Notch modulates Runx2 activity and exerts biphasic effects: early activation preserves the progenitor pool and delays differentiation, whereas later signaling promotes chondrocyte hypertrophy and osteogenic maturation.^101,102^ Fracture studies suggest that increased Notch1 signaling in periosteal osteochondroprogenitors reduces cartilage, increases mineralized callus formation, and improves mechanical strength. Conversely, Notch inhibition promotes cartilage persistence and reduces bone mass and strength. Further studies also indicate that Notch components are upregulated during both endochondral fracture healing and intramembranous defect repair, with expression decreasing during chondrogenesis but persisting in osteoblast-lineage cells. Spatially, this activity localizes to periosteal fronts, where it helps couple cartilage resorption to bone deposition.^101,103^ In ECs, Notch signaling also regulates vascular sprouting and angiocrine signaling, coordinating vascular invasion with bone formation. This vascular role intersects with BMP regulation through endothelial Notch-dependent angiocrine factors such as noggin, illustrating direct crosstalk between morphogen systems.^104,105^ Together, Hedgehog and Notch signaling appear to help regulate the tempo and fidelity of the cartilage-to-bone transition during fracture repair and may help couple skeletal maturation to periosteal and vascular organization.

### Vascularization and osteoangiogenic coupling

**Fig. 4:**
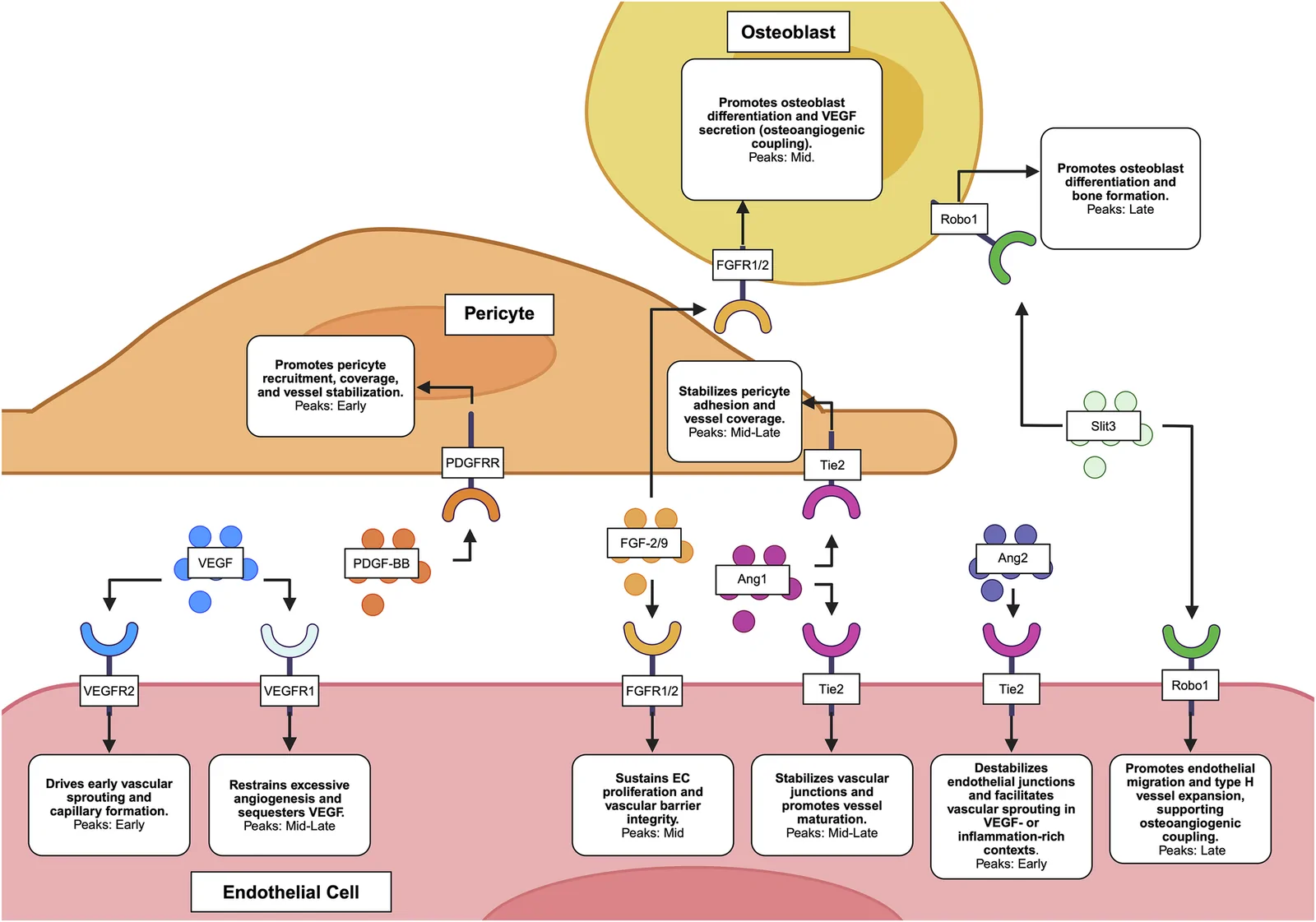
Osteoangiogenic coupling via endothelial, pericyte, and osteoblast signaling. This figure illustrates the temporal coordination of angiogenic and osteogenic signals that couples vascular invasion to bone formation during fracture healing. Three key cell types exchange paracrine cues through VEGF, PDGF, FGF, angiopoietin, and Slit/Robo axes to synchronize vascular sprouting in the early phase, stabilization in the mid phase, and maturation in the late phase with osteoblast recruitment and differentiation. Peak labels indicate relative activity peaks and should not be interpreted as exclusive windows of pathway activity. Arrows indicate ligand–receptor relationships and downstream effects. VEGF acts predominantly through endothelial VEGFR2 to promote vascular sprouting and capillary formation, whereas VEGFR1 modulates VEGF availability and limits excessive angiogenic signaling. PDGF-BB signaling promotes recruitment and stabilization of pericyte coverage primarily through PDGFRβ on pericytes and other mural cells, thereby indirectly supporting vessel maturation. Ang2, produced by endothelial cells, acts mainly through endothelial Tie2 in VEGF- or inflammation-rich contexts to destabilize vessels and facilitate sprouting, whereas Ang1 derived from pericytes, stromal cells, or osteoblast-lineage cells acts predominantly on endothelial Tie2 to promote junctional integrity, vascular quiescence, and vessel maturation. Osteoblast-derived Slit3 can signal through Robo1 on endothelial cells to promote endothelial migration and type H vessel expansion, thereby supporting osteoangiogenic coupling, and through Robo1 on osteoblasts to promote osteogenic differentiation and bone formation. The scheme emphasizes principal endogenous ligand–receptor relationships with the clearest cell-type assignments; context-dependent direct effects of VEGF on osteoblast-lineage cells and osteogenic effects reported for the engineered Ang1 variant COMP-Ang1 are discussed in the text but are not depicted. Note: Osteoclasts and pre-osteoclasts, which secrete PDGF-BB and directly contribute to osteoangiogenic coupling by recruiting vessels to resorption sites, are not depicted; their role is described in the text (Section “PDGF and FGF Pathways”). Created with BioRender.com

A functional vascular network is essential for fracture repair because angiogenesis and osteogenesis are tightly coupled (Fig. 4).^11,106,107^ ECs, pericytes, osteoblast-lineage cells, and osteoclast-lineage cells exchange paracrine and mechanical cues that coordinate vascular invasion with matrix deposition, mineralization, and remodeling. Specialized type H vessels and vessel-stabilizing pericytes suggest that vascular identity and organization, not vessel density alone, influence skeletal regeneration.^35,36^ However, evidence for type H specialization and some angiocrine mechanisms still comes mainly from broader skeletal biology and preclinical fracture models.

#### VEGF/HIF pathway

Post-fracture hypoxia stabilizes hypoxia-inducible factor-1α (HIF-1α) and may also stabilize HIF-2α in MSCs, ECs, and macrophages. Stabilized HIFs dimerize with HIF-1β and induce target genes, including VEGF, SDF-1, and glycolytic enzymes.^77,107^ VEGF released into the fracture environment binds two principal receptors on ECs. VEGF receptor 2 (VEGFR2) mediates the dominant proangiogenic effects, including endothelial proliferation, migration, tube formation, and vascular permeability, through PI3K–Akt and ERK/MAPK signaling. VEGF receptor 1 (VEGFR1) acts mainly as a high-affinity modulatory receptor, functioning as a ligand trap and weaker signaling regulator of VEGF output.^108–110^ VEGFR2 expression increases during callus formation and peaks during callus maturation, whereas VEGFR1 follows a comparatively stable monophasic pattern after the initial phase.^111^ HIF/VEGF signaling is further amplified by pro-inflammatory cytokines and morphogens, integrating immune, metabolic, and morphogenetic inputs with osteogenic differentiation. In osteoblasts, VEGF also enhances differentiation and can promote osteoclast recruitment and activity, thereby coupling bone formation to osteoclastic remodeling.^33,112^ As perfusion and oxygenation are restored, HIF degradation lowers VEGF expression, attenuating sprouting and promoting vessel maturation.^107^ The HIF/VEGF axis therefore links hypoxia to progenitor recruitment, vascular entry into the callus, and metabolic adaptation during early repair. Insufficient signaling impairs vascular invasion, whereas prolonged or poorly resolved activation may favor immature or disorganized vascular networks.^33,107^ Therapeutic benefit therefore depends more on supporting timely vascular entry and maturation than on simply increasing angiogenesis.

#### PDGF and FGF pathways

Following VEGF-driven sprouting, platelet-derived growth factors (PDGFs) and fibroblast growth factors (FGFs) promote vascular stabilization and osteogenic coupling. Platelet-derived growth factor-BB (PDGF-BB), secreted by platelets, ECs, macrophages, and pre-osteoclasts, binds platelet-derived growth factor receptor beta (PDGFRβ) primarily on pericytes and other mural cells, recruiting and retaining them at sites of active remodeling. PDGF signaling activates PI3K–Akt and ERK/MAPK pathways to promote proliferation, migration, and survival, thereby supporting vessel coverage and stability. Recruited mural cells and adjacent ECs then provide stabilizing cues, including VEGF, TGF-β, and Notch-related endothelial–perivascular crosstalk, which may reinforce endothelial quiescence and vessel maturation.^113–115^ FGF-2, released from damaged ECM and inflammatory cells, activates fibroblast growth factor receptors 1 and 2 (FGFR1/2) on ECs and osteoblasts. FGF-9, secreted by osteoblasts and chondrocytes, acts mainly on osteoblasts. These ligands activate ERK/MAPK, PI3K–Akt, and phospholipase Cγ (PLCγ) signaling to support endothelial integrity and osteoprogenitor differentiation. FGF-2 promotes early sprouting, whereas FGF-9 has been shown in preclinical skeletal repair models to link angiogenesis to osteogenesis by stimulating osteoblast proliferation and VEGF secretion. FGF signaling also interacts with the PDGF–PDGFRβ axis, with vascular models suggesting synergistic effects on mural-cell recruitment and maturation.^116–118^ PDGF and FGF signaling therefore support vascular function through mural-cell coverage, endothelial integrity, perfusion, and osteogenic competence rather than vessel density alone. This may help explain why purely pro-sprouting strategies often fail to improve repair quality.

#### Angiopoietin/Tie2 pathway

The transition from VEGF-driven sprouting to stabilized vasculature is partly regulated by angiopoietins (Ang) and their Tie2 receptor. Angiopoietin-2 (Ang2), secreted by ECs during early angiogenesis, often destabilizes vessels in the presence of VEGF or inflammatory cues, thereby facilitating endothelial migration and invasion into the cartilaginous callus. As repair progresses, angiopoietin-1 (Ang1), secreted by pericytes, osteoblasts, and stromal cells, binds Tie2 primarily on ECs and activates PI3K–Akt signaling to promote survival, junctional integrity, and quiescence. By reinforcing vascular endothelial cadherin (VE-cadherin) junctions and suppressing permeability, Ang1 cooperates with PDGF-BB to support vessel maturation.^114,119,120^ Angiopoietins may also influence osteogenesis. The chimeric Ang1 variant COMP-Ang1 has been shown to enhance BMP2-induced osteoblast differentiation and bone formation.^121^ In mural-cell biology, Tie2-related angiopoietin signaling may also influence cell motility and vessel remodeling, although the fracture-specific relevance of this mechanism is less clearly established. The balance between Ang2 and Ang1 is thought to help shift the callus from active sprouting toward a stable vascular network that supports hard callus formation and mineralization.^114,115^ This transition from vascular plasticity to vascular stability helps determine whether the callus develops into a mature perfused network or remains in an immature vascular state, with consequences for oxygen delivery, matrix mineralization, and soft-to-hard callus conversion.

#### Slit/Robo signaling and type H vessels

During mid-to-late repair, the Slit/Robo guidance system may contribute to vascular patterning and expansion of the osteoangiogenic capillary network. In broader skeletal and vascular literature, Slit3 secreted by osteoblasts and Slit2 secreted by ECs act through Roundabout receptors (Robo1/4). In ECs, Slit2-associated Robo4 signaling has been reported to reinforce VE-cadherin junctions and counteract VEGF-induced permeability, although the precise receptor interaction remains debated. More broadly, Slit/Robo signaling regulates endothelial polarity, migration, and vascular patterning through Rho-family GTPases and srGAP-linked pathways.^122,123^ Osteoblast-derived Slit3 also has been linked to Robo1-dependent endothelial responses and may contribute to expansion of type H vessels, a specialized capillary subtype located at osteogenic fronts.^35,122^ These vessels couple angiogenesis to bone formation through angiocrine signaling: high endothelial Notch signaling in type H vessels induces noggin secretion, which tunes local BMP activity and balances osteoprogenitor expansion with differentiation.^35,105^ In parallel, Slit3–Robo1 signaling in osteoblasts has been proposed to interact with β-catenin to enhance osteogenic gene expression directly. This reciprocal signaling has been reported at bone–cartilage interfaces, helping maintain coupling between vascular invasion and matrix mineralization.^122^ Although Slit/Robo signaling and type H vessel specialization have been defined mainly in experimental systems, they suggest that vascular identity, angiocrine output, and anatomical patterning may be as important as vessel abundance for successful bone regeneration. Regenerative failure may therefore reflect loss of osteogenic vascular identity rather than insufficient angiogenesis alone.

### Mechanotransduction networks

Mechanical forces generated by load-bearing, micromotion, and ECM deformation are important influences on fracture repair. Mechanotransduction systems translate these cues into biochemical signals that align osteogenic, chondrogenic, angiogenic, and remodeling responses with the evolving mechanical environment. Controlled strain supports callus formation and maturation, whereas instability, stress shielding, or prolonged unloading can impair callus organization and progression to union.^124^

#### Integrin/FAK/Hippo/YAP mechanotransduction cascade

The integrin–FAK–Hippo–YAP/TAZ axis translates mechanical strain into adaptive regenerative programs. When α/β integrins bind ECM proteins and cluster under tension, they activate FAK, which engages PI3K–Akt, ERK/MAPK, and RhoA/ROCK signaling to coordinate downstream cellular responses.^125^ FAK influences transcription through converging mechanosensitive pathways. Through RhoA/ROCK, it increases actin cytoskeletal tension and suppresses Hippo-dependent restraint of YAP/TAZ, promoting their nuclear translocation. This pathway involves inhibition of core Hippo kinases, including MST1/2 and LATS1/2, thereby enabling YAP/TAZ accumulation in the nucleus. Activated YAP/TAZ then bind TEAD transcription factors to induce gene programs linked to proliferation, survival, and osteogenic commitment. Crosstalk with Wnt and PI3K–Akt–mTOR signaling further reinforces these anabolic and osteogenic responses, including nuclear β-catenin accumulation.^125,126^ In MSCs, mechanical loading shapes fate decisions: Rho-GTPase-dependent durotaxis directs migration toward stiffer matrices, whereas sustained cyclic or static loading maintains nuclear YAP and β-catenin accumulation and promotes osteogenic commitment and collagen synthesis.^127^ In ECs, FAK regulates cell shape and VE-cadherin dynamics to support vessel stability.^128^ Early YAP/TAZ activation supports progenitor expansion, whereas sustained loading in the hardening callus promotes mineralization, with activity greatest at periosteal fronts. By contrast, unloading and reduced matrix tension drive YAP/TAZ sequestration in the cytoplasm through reactivation of Hippo kinases, suppressing osteogenic gene expression and contributing to impaired callus formation.^129^ These findings help explain why fracture repair is highly sensitive to fixation stiffness, interfragmentary motion, and rehabilitation strategy. They also suggest why biologic therapies targeting this axis may perform inconsistently across fixation conditions: FAK–YAP/TAZ signaling remains inseparable from the mechanical context in which cells interpret strain, stiffness, and matrix tension.

#### Osteocyte and canalicular mechanotransduction

**Fig. 5:**
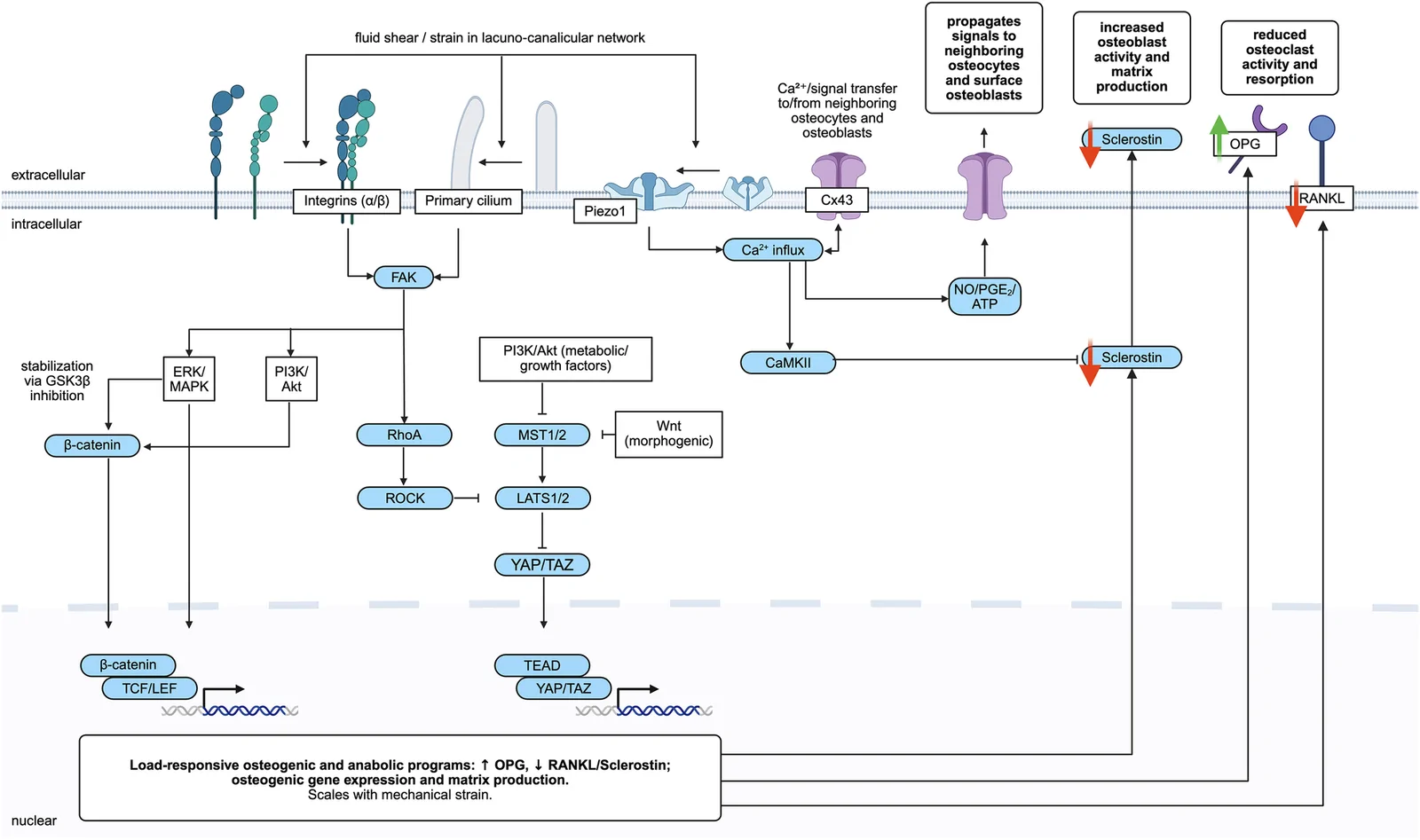
Osteocyte mechanotransduction couples mechanical loading to bone formation. This figure illustrates how osteocytes sense mechanical strain through the lacuno–canalicular network and translate physical signals into coordinated anabolic and anti-catabolic programs. Mechanisms are shown primarily as osteocyte mechanobiology pathways relevant to callus maturation and remodeling; not every molecular interaction should be interpreted as fracture-callus specific. Fluid shear and strain activate Piezo1 ion channels, integrins, and the primary cilium, with Piezo1 promoting rapid Ca²⁺ influx, integrin-associated signalling recruiting FAK as a central signalling hub, and the primary cilium contributing through ciliary mechanosensitive signalling pathways. FAK engages parallel cascades: ERK/MAPK and PI3K/Akt pathways that stabilize and activate β-catenin via GSK3β inhibition and nuclear translocation; and RhoA/ROCK-mediated inhibition of MST1/2–LATS1/2, enabling YAP/TAZ nuclear accumulation and TEAD-dependent gene expression. Concurrently, Ca²⁺ influx activates CaMKII and other calcium-sensitive pathways, which contribute to rapid intracellular inhibition and lysosomal degradation of sclerostin protein and stimulate production of paracrine mediators, including NO, PGE₂, and ATP. Cx43 gap junctions support direct intercellular transfer of Ca²⁺-related signals and other small molecules between neighboring osteocytes and bone-surface cells, whereas Cx43 hemichannels contribute to extracellular mediator release, particularly PGE₂. The net outcome is load-responsive osteogenic gene expression, including increased OPG and decreased RANKL/sclerostin, together with enhanced matrix production and suppression of osteoclast activity, with responses influenced by strain magnitude, frequency, and mechanical context. Red arrows indicate low expression and therefore low protein levels; green arrows indicate high expression and therefore high protein levels. Created with BioRender.com

Osteocytes are key mechanosensory cells in bone (Fig. 5), detecting strain and fluid shear through the lacuno–canalicular network (LCN), a fluid-filled system that connects osteocytes to one another and to bone-surface cells. Although classically associated with remodeling in intact bone, osteocytes also contribute to fracture repair by translating local strain into anabolic and antiresorptive signals during callus maturation and remodeling. Mechanical stimulation is sensed through integrin-based adhesions and the primary cilium, with FAK acting as a central mechanotransductive node. Activated FAK stimulates ERK/MAPK signaling and promotes nuclear localization of YAP/TAZ and β-catenin. These responses support transcriptional programs associated with increased osteoprotegerin (OPG) and IGF-1, reduced receptor activator of nuclear factor κB ligand (RANKL), and suppression of inhibitors such as sclerostin, a secreted Wnt antagonist that normally restrains bone formation.^130^ Mechanical loading can activate mechanosensitive ion-channel signaling, including Piezo1-dependent Ca²⁺ influx, while downstream calcium-sensitive pathways such as CaMKII contribute to regulation of bone mass and quality.^131,132^ Elevated intracellular Ca²⁺ and CaMKII activity are associated with rapid lysosomal degradation of sclerostin, relieving Wnt inhibition on neighboring osteoblasts more quickly than transcriptional repression alone.^132,133^ In parallel, Ca²⁺ elevation stimulates production of nitric oxide (NO), prostaglandin E₂ (PGE₂), and adenosine triphosphate (ATP). Cx43 gap junctions support direct intercellular communication between neighboring osteocytes and bone-surface cells, whereas Cx43 hemichannels contribute to extracellular mediator release, including PGE₂. NO diffuses locally, while extracellular ATP activates purinergic signaling. NO and PGE₂ support osteoblast survival and activity, modulate OPG/RANKL expression, and promote local vasodilation, whereas ATP helps propagate additional osteogenic and immunomodulatory responses.^43,134^ These mechanisms help explain why mechanical environment and biological healing quality remain tightly linked even after early inflammation has subsided, and why radiographic union does not necessarily indicate full mechanical recovery. Their translational appeal lies in load-adaptive regeneration, but effective therapeutic use will likely require pharmacologic modulation to be paired with an appropriate loading environment.

### Matrix turnover and coupling

Matrix turnover and osteoclast–osteoblast coupling characterize the late remodeling stage of fracture repair, during which provisional cartilage and woven bone are progressively replaced by mechanically competent lamellar bone. This process depends on coordinated proteolysis, receptor–ligand signaling, and mechanical feedback that reshape the callus and restore skeletal integrity.^135,136^

#### Proteolytic remodeling axes

Balanced matrix turnover is important for the efficient conversion of the disorganized callus into load-bearing bone. This process is driven by matrix MMPs and a disintegrin and metalloproteinase with thrombospondin motifs (ADAMTS) proteases, whose expression is regulated by growth factors, cytokines, and mechanical cues.^137^ MMP-9, produced by osteoclasts, immune cells, and ECs, facilitates vascular invasion and cartilage removal.^138^ MMP-13, produced by hypertrophic chondrocytes and osteoblasts, degrades collagen and supports the cartilage-to-bone transition.^139^ MMP-2, produced by osteoblasts and fibroblasts, contributes to later matrix refinement.^140^ MMP activity is tightly controlled by tissue inhibitors of metalloproteinases (TIMPs), produced by mesenchymal and stromal cells in response to growth factors and cytokines, which limit excessive degradation.^137,141^ Complementing this system, ADAMTS4/5, produced by chondrocytes and fibroblasts, degrade proteoglycans such as aggrecan and thereby support coordinated cartilage resorption and restoration of lamellar bone architecture.^142,143^ Insufficient proteolysis can delay vascular invasion and cartilage clearance, whereas excessive or mistimed activity may destabilize nascent repair tissue.^144^ Fracture-healing failure may therefore reflect defective matrix turnover and tissue conversion, not only deficient osteogenesis.

#### Resorption-formation coupling axes

**Fig. 6:**
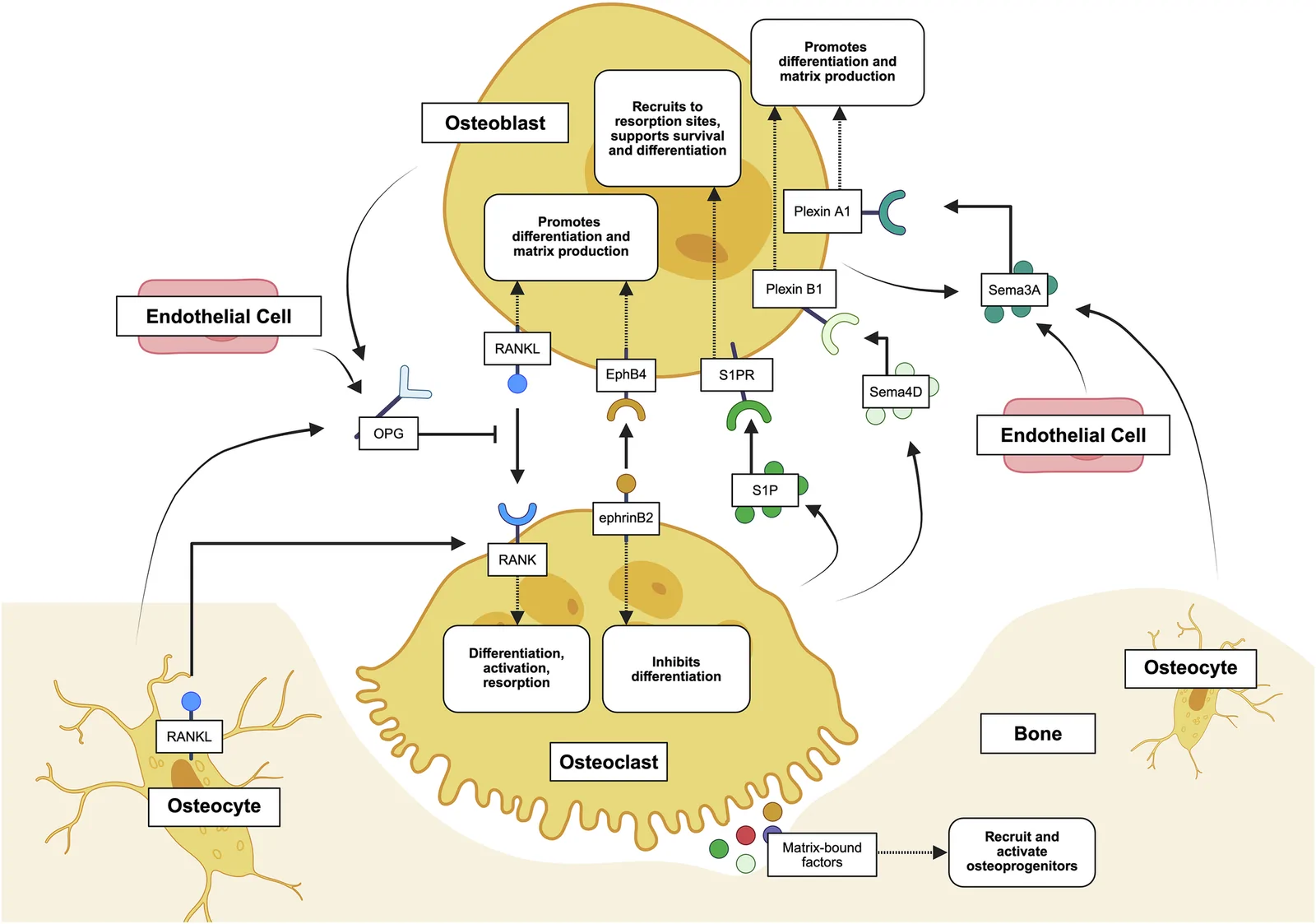
Resorption-formation coupling during bone remodeling. This figure illustrates signaling axes that coordinate osteoclast-mediated resorption with osteoblast-driven bone formation. The RANKL/OPG/RANK axis is central: RANKL, produced mainly by osteoblasts, osteocytes, stromal cells, and T cells, activates osteoclastogenesis via RANK, while OPG, secreted by osteoblasts, osteocytes, and endothelial cells, sequesters RANKL to limit resorption. Bidirectional EphB4/ephrinB2 signaling refines this balance: forward signaling promotes osteoblast matrix deposition, while reverse signaling suppresses osteoclast differentiation. Soluble mediators further modulate coupling: S1P recruits osteoblasts to resorption sites and supports survival and differentiation via S1PR1/3 receptors; Sema3A, produced primarily by osteoblasts and osteocytes, with possible context-dependent endothelial contribution, inhibits osteoclastogenesis while enhancing osteoblast differentiation through the Nrp1/Plexin A1 receptor complex; and Sema4D from osteoclasts restrains bone formation via Plexin B1. Matrix-bound factors, including TGF-β and IGF-1, are released during resorption and recruit and activate osteoprogenitors, directly linking catabolism to anabolism. Osteocyte mechanotransduction (Fig. 5) further refines this equilibrium to ensure remodeling scales with mechanical demand. Straight arrows point from ligand to the receptor it binds to; straight dashed arrows point from ligand to effect; and bent arrows indicate secretion. Created with BioRender.com

Bone remodeling depends on coordinated communication between osteoclasts and osteoblast-lineage cells through receptor–ligand, paracrine, and contact-mediated systems (Fig. 6). Central to this process are receptor activator of nuclear factor κB (RANK), its ligand RANKL, and the decoy receptor osteoprotegerin (OPG). RANKL, produced by osteoblasts, osteocytes, stromal cells, and T cells, binds RANK on osteoclast precursors and activates NF-κB, nuclear factor of activated T cells 1 (NFATc1), and MAPK signaling to drive osteoclast differentiation and resorption. OPG, secreted by osteoblasts, osteocytes, and ECs, sequesters RANKL and limits osteoclastogenesis. The RANKL/OPG ratio therefore determines the net direction of turnover and is itself shaped by Wnt/β-catenin signaling, estrogen status, and inflammatory cytokines.^145^ Direct cell-to-cell coupling also contributes to the shift from resorption toward formation. Forward signaling through ephrin type-B receptor 4 (EphB4) on osteoblasts promotes matrix deposition, whereas reverse signaling through ephrinB2 on osteoclasts, their precursors, and other stromal cells suppresses NFATc1-dependent osteoclastogenesis.^146^ Soluble mediators further refine this balance. Sphingosine-1-phosphate (S1P), secreted by osteoclasts and ECs, recruits osteoblasts to resorption sites and supports their survival through S1P receptor (S1PR) signaling. Semaphorin 3 A (Sema3A), produced primarily by osteoblasts and osteocytes, with possible context-dependent endothelial contribution, inhibits osteoclast differentiation and enhances osteoblast activity by binding to the Neuropilin-1 (Nrp1) co-receptor, which signals through the transmembrane Plexin A1 receptor complex. By contrast, osteoclast-derived Semaphorin 4D (Sema4D) restrains bone formation through Plexin-B1 signaling in osteoblasts.^136^ Acidic osteoclastic resorption also releases matrix-stored factors such as TGF-β and IGF-1, which recruit and stimulate osteoprogenitors and thereby couple catabolism to anabolism.^147^ Osteocytes further fine-tune this equilibrium by dynamically adjusting RANKL/OPG expression, potentially through FAK–YAP/TAZ-dependent mechanotransduction, so that remodeling remains aligned with mechanical demand.^129,148^ These interactions underscore that osteoclasts are not merely terminal resorptive cells, but important organizers of regenerative coupling during fracture remodeling. In fracture healing, both premature suppression and excessive persistence of resorption may impair conversion of woven callus into mature load-bearing tissue.^135,136,140^ This complicates the use of standard bone-active drugs that suppress osteoclast activity while also removing resorptive and signaling events required for structural remodeling and subsequent bone formation.

Together, these pathways show that successful fracture healing depends on coordinated transitions between inflammatory activation, progenitor recruitment, vascular restoration, lineage specification, mechanoadaptation, and matrix remodeling. Disruption at any stage can redirect repair toward delayed union, nonunion, or biologically incomplete healing, setting the stage for the pathological contexts discussed in Section “Disruption of fracture healing: modulators and mechanisms”.

## Disruption of fracture healing: modulators and mechanisms

Fracture healing can be disrupted by systemic and local factors that impair the coordinated progression from inflammation and progenitor recruitment to vascularization, matrix conversion, and remodeling. These disruptions increase the risk of delayed union, nonunion, and mechanically compromised repair tissue.^149,150^ The following sections define the principal phenotypes of impaired healing and examine the modulators that bias repair toward failure.

### Clinical phenotypes

Impaired fracture healing spans a spectrum defined by both the tempo and quality of repair. The principal clinical endpoints are delayed union and nonunion, which differ in radiographic progression and underlying biology.^151^ Delayed union refers to a fracture that shows limited radiographic or clinical progression, typically after approximately 3–6 months, often with some evidence of ongoing biological activity within the callus.^151,152^ Nonunion denotes failure of progressive healing and is commonly diagnosed after approximately 6–9 months. However, some guidelines, including those of the US Food and Drug Administration (FDA), use a threshold of at least 9 months from injury with no radiographic progression over a 3-month interval. Radiographically, nonunions are often classified as hypertrophic, with abundant callus but absent bridging, suggesting preserved biological activity but inadequate mechanical stability; atrophic, with minimal callus and impaired vascularity or cellular activity; or oligotrophic, with intermediate features. Long-standing cases may progress to pseudarthrosis, characterized by a mobile fibrous or synovial-like interface.^150–152^ Beyond these formal clinical categories, a conceptually important outcome is mechanically inferior union. In such cases, continuity is achieved, but the repaired segment remains structurally deficient, with low mineral density, abnormal microarchitecture, or reduced strength. This may be subtle on routine imaging yet could plausibly contribute to poorer long-term mechanical performance, particularly where regenerate quality remains compromised.^153,154^

### Modulators of fracture healing

Fracture repair is shaped by systemic and local modulators (Table 1), together with genetic and epigenetic factors that influence individual susceptibility. As a synthesis of the mechanisms outlined above, these modifiers act through the signaling and cellular programs outlined in Section “Signaling networks and regulatory axes in fracture healing”, including inflammatory resolution, progenitor recruitment, osteoangiogenic coupling, lineage progression, mechanotransduction, and remodeling. They therefore influence not only the likelihood of healing failure, but also the phenotype of failure and the context in which biologic augmentation may succeed or fail.

**Table 1:** Modulators of impaired fracture healing mapped to dominant mechanisms, repair-phase effects, and clinical phenotype

Modulator	Cellular and molecular effects	Impact on repair phases	Clinical phenotype
Aging	Senescence-associated inflammation, reduced MSC osteogenic capacity, altered Wnt/β-catenin, IGF-1, and BMP/TGF-β signaling, impaired mechanotransduction, and reduced angiogenic responsiveness.^155–157^	Slower callus mineralization, weaker vascular and anabolic responses, and reduced bone formation.^157,158,163,164^	May bias repair toward delayed union, low-callus or atrophic-appearing repair, and reduced mechanical quality of the regenerate, particularly with additional local or metabolic stressors.^157,162,163^
Sex hormone deficiency	Altered osteoblast–osteoclast balance, RANKL/OPG imbalance, oxidative stress, NF-κB activity, inflammatory cytokines, and increased Wnt inhibition through sclerostin and related antagonists.^164–169^	Callus formation may be delayed, inflammatory and remodeling phases can be altered, and mineral accrual or mechanical recovery may remain incomplete in estrogen-deficient models.^170,171^	May contribute to impaired repair quality, especially when combined with mechanical or metabolic stress.^151,152,164^
Diabetes	AGE/RAGE activation, oxidative stress, impaired osteoblast differentiation, adipogenic MSC drift, reduced VEGF expression, and microvascular dysfunction.^173,174,176^	Hypovascular, smaller, less mineralized callus with delayed remodeling and increased chondrocyte and endothelial apoptosis.^173,175,176^	Associated with increased delayed union and nonunion; healing failure may show low-callus or atrophic-appearing features, particularly with vascular dysfunction, infection risk, or prolonged inflammation.^151,152,173^
Smoking	Oxidative stress, impaired MSC and osteoblast proliferation and differentiation, vascular dysfunction, and disrupted Wnt/β-catenin and BMP/TGF-β signaling.^177,178^	Reduced callus perfusion and vascularity, impaired osteoangiogenic coupling, smaller callus, reduced mineralization, and weaker mechanical properties.^177,178^	Associated with increased risk of delayed union and nonunion and with impaired callus formation and reduced mechanical integrity.^179,180^
NSAID exposure	COX-2 inhibition with reduced PGE₂ signaling; impaired chondrogenic differentiation and downregulation of endochondral regulators such as TGF-β3.^182–184^	Blunted early inflammatory/angiogenic signaling, reduced cartilage formation, and impaired callus formation or vascularity in experimental settings.^182–184^	May increase delayed union or nonunion risk depending on dose, duration, timing, patient age, fracture type, and baseline healing risk; clinical evidence remains mixed.^185–187^
Polytrauma/systemic injury	Sustained systemic inflammation with elevated TNF, IL-6, and DAMP signaling.^188^	Smaller, less mineralized, mechanically weaker callus and impaired coupling of inflammation, regeneration, and remodeling in preclinical models.^189^	Associated with increased risk of delayed union and nonunion in severe-trauma models and selected clinical cohorts; fixation timing may influence risk but is confounded by injury severity.^152,189,190^
Traumatic brain injury	Altered neuroimmune, neuroendocrine, and osteogenic signaling, including altered fracture-hematoma inflammation, sympathetic tone-mediated marrow myelopoiesis, and circulating cytokine/growth-factor changes.^81,191,194,195^	Shorter time to callus formation, increased callus size, and faster radiographic healing in some models and cohorts, but variable final repair quality.^191–193^	Altered or hyper-regenerative healing phenotype; in some studies may favor exuberant callus formation, but not necessarily optimized final bone quality.^191^
Spinal cord injury	Altered neuromechanical and neuro-osseous regulation, including unloading-related osteoclastic dominance, SCI-induced osteopenia, impaired chondro-osseous junction function, altered angiogenesis, and context-dependent effects of spasticity or abnormal mechanical stimulation.^196–199^	Heterogeneous effects on callus formation, bony bridging, angiogenesis, and remodeling, with enhanced bridging in some models but impaired callus formation or vascular responses in others.^196,197^	Heterogeneous altered-healing phenotype in preclinical models, with enhanced bridging in some settings but impaired healing or mechanical quality in others; human fracture-healing evidence remains limited.^196–199^
Rheumatic disease	Chronic osteoimmune imbalance with persistent inflammatory cytokine signaling, enhanced osteoclastogenesis, and impaired angiogenesis or osteogenic progression.^200–202^	Poor callus formation, diminished angiogenesis, disrupted remodeling, and persistent inflammatory burden in experimental inflammatory-disease models.^200^	May increase risk of delayed union, nonunion, and poor remodeling; clinical evidence is suggestive but not uniform and may be shaped by both disease activity and treatment exposure.^200–202^
Nutritional deficiency	Insufficient protein, calcium, or vitamin D availability may compromise matrix production, osteoblast function, and mineralization.^203,204^	May compromise reparative callus formation and later mineralization/remodeling when protein, calcium, or vitamin D deficiency is present.^203,204^	Metabolic and endocrine abnormalities have been identified in selected nonunion patients, but causality has not been established, and the effect of nutritional supplementation on fracture union remains uncertain.^203,204^
Heavy alcohol exposure	Alcohol exposure impairs fracture-callus formation and endochondral repair in experimental models.^152,205^	Reduced callus volume and strength and disrupted endochondral ossification in experimental models.^205^	Plausibly contributes to delayed repair and reduced mechanical quality of the regenerate after heavy exposure; human nonunion evidence remains limited.^152,205^
Microbiome dysbiosis	Altered osteoimmune signaling and T-cell phenotypes, with broader effects on bone-regulatory immune programs.^206^	May impair callus formation through altered osteoimmune signaling in preclinical models.^206^	Potential contribution to delayed or impaired repair; human fracture-healing data remain limited.^206^
Environmental toxins	Heavy-metal exposure may disrupt osteoblast–osteoclast activity, bone remodeling balance, and matrix quality.^207–209^	Direct phase-level human fracture-healing evidence is limited.^207–209^	Could plausibly impair healing quality; human evidence more consistently links exposure to lower BMD and fragility fracture risk than to defined delayed-union or nonunion endpoints.^207–209^
Mechanical instability/excessive motion	Excessive IFM and high strain, especially shear or bending, disrupt mechanically guided callus progression.^129,210–212^	Excessive shear or high-strain motion delays bridging despite biological callus activity and may prevent progression to union.^210,213,214^	Classically associated with hypertrophic nonunion and possible pseudarthrosis if persistent.^150–152,214^
Excessive rigidity/stress shielding	Minimal motion favors primary/direct healing when reduction, cortical contact, and absolute stability are achieved; stress shielding or insufficient mechanical stimulation may attenuate callus formation.^215^	Reduced callus formation or mechanically suboptimal remodeling, particularly when biological capacity is impaired.^210,215,216^	May contribute to delayed healing or mechanically inferior union in biologically compromised settings.^210,215,216^
Persistent inflammation/infection	Sustained TNF, IL-1β, and IL-6 signaling maintains pro-inflammatory macrophage activity, increases resorption, and suppresses osteogenic differentiation and angiogenic progression.^10,150,217^	Arrested transition from inflammation to repair, impaired vascular integration, and fibrotic or disorganized repair tissue.^10,150,218^	May result in fracture-related infection and nonunion; radiographic callus morphology is variable and does not independently establish infection status or regenerative potential.^10,150,217,218^
Open fracture/soft-tissue damage	Loss of regional perfusion, hematoma stability, periosteal progenitor supply, and local paracrine cues, with tissue necrosis and increased contamination burden.^219,220^	Blunted periosteal callus formation, prolonged inflammation, impaired endothelial ingrowth, and reduced bridging.^219,223,224^	Can predispose to delayed union, nonunion, or fracture-related infection, particularly in high-energy injury patterns.^219,221–224^
Segmental bone loss/critical-sized defect	Loss of bone continuity and osteogenic interface, loss of cortical contact and load sharing, mechanically unfavorable motion depending on fixation and gap geometry, and limited angiogenic penetration across the defect.^225^	Bridging callus formation and maturation are limited by gap size, mechanical conditions, and insufficient vascularized tissue volume; repair becomes dependent on restoration of mechanical continuity and biologic volume.^225–227^	Reconstruction-dominant segmental defect nonunion; often excluded from union-acceleration trials or analyzed separately.^226^

This table summarizes the modulators discussed in Section “Disruption of fracture healing: modulators and mechanisms” and links them to their main cellular or molecular effects, their predominant impact on repair progression, and the associated clinical phenotype. The table is intended as a simplified framework rather than an exhaustive mechanistic map. Genetic and epigenetic determinants are not included because they act as cross-cutting susceptibility modifiers rather than discrete systemic or local modulators

*AGE* advanced glycation end product, *BMD* bone mineral density, *BMP* bone morphogenetic protein, *COX-2* cyclooxygenase-2, *DAMP* damage-associated molecular pattern, *FAK* focal adhesion kinase, *IFM*, interfragmentary motion, *IGF-1* insulin-like growth factor-1, *MSC* mesenchymal stromal/stem cell, *NF-κB* nuclear factor-κB, *NSAID* nonsteroidal anti-inflammatory drug, *OPG* osteoprotegerin, *PGE*₂ prostaglandin *E*₂, RA, rheumatoid arthritis, *RAGE* receptor for AGE, *RANKL* receptor activator of nuclear factor κB ligand, *SASP* senescence-associated secretory phenotype, *SCI* spinal cord injury, *TBI* traumatic brain injury, *TGF-β* transforming growth factor beta, *TNF* tumor necrosis factor, *VEGF* vascular endothelial growth factor, *Wnt* Wingless/Integrated

#### Systemic modulators

Systemic factors influence fracture healing through endocrine, metabolic, inflammatory, and vascular mechanisms that alter the repair environment. Rather than acting through a single pathway, they perturb multiple signaling systems in parallel and can shift repair toward delayed union, nonunion, or mechanically inferior regeneration.

##### Aging

Aging is associated with accumulation of senescent cells in mesenchymal and osteolineage compartments, including MSCs, osteoprogenitors, osteoblasts, and osteocytes. These cells can acquire a senescence-associated secretory phenotype (SASP) characterized by inflammatory cytokines such as IL-1, IL-6, and TNF, as well as proteases and growth factors. Within the aged bone microenvironment, SASP signaling from bone marrow MSCs, osteoblastic cells, and senescent osteocytes has been linked to enhanced osteoclastogenesis, including through RANKL-mediated pathways.^155^ In parallel, canonical Wnt/β-catenin and IGF-1 signaling decline, BMP/TGF-β activity becomes dysregulated, and both intramembranous and endochondral bone formation are reduced.^156,157^ Aging has also been associated with reduced skeletal responsiveness to mechanical and regenerative cues, together with diminished angiogenic responsiveness during fracture repair.^158^ Frailty and sarcopenia may further reduce mechanical loading and trophic support.^159–161^ Collectively, these changes slow callus mineralization, weaken vascular and anabolic responses, and may bias repair toward delayed union, low-callus or atrophic-appearing repair, and mechanically inferior repair, even when union is ultimately achieved, particularly in the presence of additional local or metabolic stressors.^157,162,163^

##### Sex hormones

Sex steroids regulate skeletal remodeling and may influence fracture repair by affecting osteoblast–osteoclast balance, mechanotransduction, and matrix quality.^164^ Estrogen signaling through estrogen receptors alpha and beta (ERα/β) in osteoblasts, osteoclasts, and osteocytes suppresses resorption by reducing RANKL and increasing OPG. It also supports osteoblast survival and matrix production through crosstalk with Wnt/β-catenin, PI3K–Akt, and COX-2/PGE₂-dependent mechanosensitive pathways.^165–167^ Estrogen deficiency, whether postmenopausal or hypogonadal, increases oxidative stress and NF-κB activity, elevates TNF, IL-1, and IL-6, impairs osteoblast and stromal-cell function, and promotes osteoclastogenesis. It also increases osteocyte-derived sclerostin and other Wnt antagonists, thereby weakening load-induced anabolic responses and shifting remodeling toward net bone loss.^165,166,168,169^ In estrogen-deficient fracture models, callus formation may be delayed, inflammatory and remodeling phases can be altered, and mechanical recovery may remain incomplete.^170,171^ In men, testosterone deficiency and reduced androgen receptor signaling are associated with reduced bone formation and altered remodeling balance, including RANKL/OPG-related effects.^172^ Clinically, low sex-hormone states may therefore contribute not only to fracture risk, but could also contribute to impaired repair quality, especially when combined with additional mechanical or metabolic stress.^151,152,164^

##### Diabetes

Chronic hyperglycemia promotes advanced glycation end product (AGE) accumulation and receptor for AGE (RAGE) activation in bone and stromal compartments, increasing oxidative stress and inflammatory signaling. These changes drive apoptosis, impair osteoblast differentiation and matrix production, and promote adipogenic drift of MSCs.^173,174^ In diabetic fracture models, these alterations are accompanied by reduced osteogenic signaling and diminished growth-factor activity, resulting in smaller, less mineralized callus and delayed remodeling.^173,175^ Diabetes-related microvascular dysfunction further compromises repair, with reduced VEGF expression, lower callus vessel density, persistent inflammatory cytokines, and increased chondrocyte and endothelial apoptosis. Together, these effects disrupt osteoangiogenic coupling.^173,176^ Diabetic fracture models show hypovascular, low bone-volume, and biomechanically weaker callus, with fibrous or adipose-tissue enrichment reported in some settings.^173,175^ Clinically, diabetes is associated with increased rates of delayed union and nonunion, and diabetic nonunions may show low-callus or atrophic-appearing features, particularly when vascular dysfunction, infection risk, or prolonged inflammation is present.^151,152,173^

##### Smoking

Cigarette smoke and nicotine impair MSC and osteoblast proliferation and osteogenic differentiation, in part through oxidative stress and disruption of key osteogenic pathways, including Wnt/β-catenin and BMP/TGF-β signaling.^177,178^ Smoking also causes vasoconstriction and endothelial dysfunction, reducing callus perfusion and vascularity and producing a smaller, less mineralized, and mechanically weaker callus, consistent with impaired osteoangiogenic coupling.^177,178^ Sustained inflammatory signaling, including NF-κB activation and increased RANKL expression, further favors osteoclastogenesis and catabolic remodeling.^177,178^ Clinically, smokers have approximately a twofold higher risk of delayed union or nonunion than nonsmokers, and smoking is consistently overrepresented in nonunion cohorts. Smoking is therefore associated with increased risk of delayed union and nonunion, often with impaired callus formation and reduced mechanical integrity.^179–181^

##### NSAIDs

Nonsteroidal anti-inflammatory drugs (NSAIDs) inhibit cyclooxygenase activity, especially COX-2, thereby reducing PGE₂ production during the early inflammatory and angiogenic phases of fracture repair. COX-2-deficient fracture models show impaired callus formation and vascularity, and experimental work indicates that PGE₂/EP4 signaling contributes to bone repair.^182,183^ In vitro, NSAIDs can inhibit chondrogenic differentiation of MSCs and downregulate regulators of endochondral ossification, such as TGF-β3, consistent with reduced cartilage formation and matrix deposition under COX inhibition.^184^ Clinically, the evidence is mixed. Some studies and earlier meta-analyses suggested an association between prolonged or high-dose NSAID use and nonunion risk,^185^ whereas more recent large-scale analyses report no consistently significant overall effect after adjustment, with pediatric data generally reassuring.^186,187^ NSAID effects on fracture healing therefore appear to depend on timing, dose, duration, and baseline healing context.

##### Polytrauma/systemic injury

Polytrauma, defined as multiple severe concurrent injuries, induces a sustained systemic stress response with prolonged inflammation and elevated TNF, IL-6, and DAMP signaling, which may disrupt callus formation and delay progression through repair in preclinical studies.^188^ Multiple-trauma fracture models show smaller, less mineralized, mechanically weaker callus and impaired coupling of inflammation, regeneration, and remodeling.^189^ In severe-trauma models and selected clinical cohorts, severe concomitant injury has been associated with delayed union or nonunion risk.^152,189,190^ Fixation strategy can further influence this risk. In experimental severe-trauma models and some clinical cohorts, damage control orthopedics with delayed conversion to definitive fixation has been discussed in relation to worse healing outcomes in selected settings, although this association is likely confounded by injury severity and physiologic instability.^190^

##### Traumatic brain injury

Traumatic brain injury (TBI) can markedly alter fracture healing and is often associated with accelerated callus formation and exuberant repair, although the magnitude of this effect varies across injury patterns, clinical cohorts, and experimental models.^191^ Preclinical and clinical studies have reported shorter time to callus formation, increased callus size, and faster radiographic healing in the setting of concomitant TBI, supporting a reproducible but heterogeneous hyper-regenerative phenotype.^191–193^ Proposed mechanisms linking concomitant TBI to altered fracture repair include altered fracture-site inflammation and hematoma biology, sympathetic tone-mediated bone marrow myelopoiesis with downstream immune-cell effects, neuroendocrine and neuropeptide signaling, and altered circulating cytokines and growth factors; however, the relative contribution of these pathways remains incompletely resolved.^81,191,194,195^ TBI may therefore amplify early reparative signaling and callus formation, but accelerated or exuberant early repair should not be assumed to produce uniformly superior final mechanical quality or optimized bone architecture.^191^

##### Spinal cord injury

Spinal cord injury (SCI) can alter fracture healing, but the direction and magnitude of its effects appear heterogeneous and are supported mainly by preclinical evidence. Experimental studies suggest that SCI-associated spasticity or abnormal mechanical stimulation can accelerate callus formation in some contexts, whereas severe SCI models with sublesional bone loss show impaired fracture healing, reduced angiogenesis, diminished callus formation, and inferior biomechanical recovery.^196,197^ These divergent findings likely reflect differences in injury severity, timing after SCI, loading environment, spasticity, vascular response, and the degree of SCI-induced osteopenia. Proposed mechanisms include unloading-related osteoclastic dominance, impaired osteoblast and chondro-osseous junction function, altered angiogenesis, and broader neuro-osseous crosstalk; however, evidence for the crosstalk concept comes in part from fracture-induced spinal-cord signaling rather than directly from SCI models.^198,199^ SCI is also associated with rapid osteoclastic resorption and deterioration of skeletal quality, which may compromise fracture repair regardless of any concurrent pro-callus stimulus.^198,199^

##### Rheumatic diseases

Inflammatory rheumatic diseases, particularly rheumatoid arthritis (RA), may impair fracture healing by imposing a chronic osteoimmune imbalance on the repair environment. Persistent inflammatory signaling promotes osteoclastogenesis, disrupts callus remodeling, and may impair angiogenesis and osteogenic progression. Experimental data strongly support this mechanism, with arthritic fracture models showing poor healing, diminished angiogenesis, reduced cartilaginous and bony callus formation, fibrotic repair tissue, persistent fracture gaps, and reduced biomechanical strength.^200^ Clinical evidence is mixed: impaired healing has been reported in selected settings, particularly femoral shaft and atypical fracture contexts, whereas other fracture types show less consistent differences.^201^ RA therapies, including conventional and biologic disease-modifying anti-rheumatic drugs, substantially modify the osteoimmune environment, but whether this improves or impairs fracture union remains insufficiently established. Current evidence on disease-modifying anti-rheumatic drugs and bone union remains limited and heterogeneous; conventional disease-modifying anti-rheumatic drugs do not clearly impair union, whereas some biologic-exposed cohorts, particularly in surgical bone-union contexts, have raised concerns about less favorable union-related outcomes or higher revision rates.^202^

##### Environmental factors

Nutritional adequacy, especially sufficient protein, calcium, and vitamin D, supports bone matrix production, osteoblast function, and mineralization. Deficiency, particularly low protein or vitamin D, has been clinically associated with prolonged healing in some cohorts, including older or hospitalized patients.^203,204^ Heavy alcohol exposure suppresses osteoblast activity and angiogenesis and impairs MSC chondrogenesis. Experimental models show reduced callus volume and strength, disrupted endochondral ossification, and impaired neovascularization, supporting a plausible link to delayed repair after heavy alcohol exposure.^152,205^ The gut microbiome may also modulate osteoimmune signaling during fracture repair. For example, in mice, antibiotic-induced depletion of the intestinal microbiota impaired fracture repair by disrupting microbiome-dependent intestinal Th17-cell expansion and trafficking to the fracture callus. However, microbiome restoration as a fracture-healing strategy remains preclinical, and human healing data are still limited.^206^ Environmental toxins such as cadmium and lead have been associated with adverse skeletal outcomes. Population-based data link increasing cadmium exposure to lower bone mineral density and increased bone resorption, while cadmium is more broadly recognized as an environmental toxicant affecting bone health. Higher lead exposure has likewise been associated with lower femoral and spinal bone mineral density and higher FRAX-estimated fracture risk.^207–209^ These exposures could plausibly impair healing quality, although the strength of evidence varies across exposures and human fracture-healing endpoint data remain limited. Overall, environmental factors should be viewed as biologically plausible modulators of repair, with evidence ranging from direct preclinical fracture-healing data to indirect skeletal and epidemiological associations.

#### Local modulators

Local modulators are factors within the fracture microenvironment that shape the mechanical, vascular, inflammatory, and structural conditions at the fracture interface. More directly than systemic modulators, they define the local bottleneck that biases healing toward a specific clinical phenotype.

##### Mechanical instability and stimulus

Mechanical conditions at the fracture site strongly govern healing outcome.^210^ Interfragmentary motion (IFM) and strain are sensed by callus and periosteal cells through mechanotransduction networks, including integrin–FAK signaling and downstream mechanosensitive pathways such as YAP/TAZ-regulated transcription.^129,211,212^ Controlled, predominantly axial IFM early after injury supports secondary healing with cartilaginous callus and endochondral ossification.^210,213^ In contrast, excessive motion and high strain, especially shear or bending, delay healing, increase nonunion risk, and are classically associated with hypertrophic nonunion, characterized by abundant callus without bridging and, if persistent, progression to pseudarthrosis.^214^ Very rigid fixation with minimal motion instead favors primary, or direct, healing with little callus, whereas stress shielding or insufficient mechanical stimulation may attenuate callus formation, particularly in settings of impaired biologic capacity.^215^ During consolidation and remodeling, physiologic loading remains important because osteocyte- and osteoblast-mediated mechanosignaling helps coordinate osteoclast activity, including through RANK/RANKL/OPG-related remodeling pathways, and supports replacement of woven callus by lamellar bone. Abnormal or insufficient loading may therefore compromise regenerate quality even when radiographic bridging is achieved.^210,216^ Mechanical conditions influence not only whether union occurs, but also how well the regenerate matures.

##### Persistent inflammation and infection

Prolonged local inflammation impairs healing by preventing timely transition from inflammatory to reparative phases.^10^ When driven by infection, necrotic tissue, dead space, or foreign-body reactions, sustained TNF, IL-1β, and IL-6 signaling maintains pro-inflammatory macrophage activity, increases resorption, and suppresses osteogenic differentiation and angiogenic progression.^10,150,217^ This reflects persistent activation of NF-κB- and JAK/STAT-linked inflammatory pathways, with downstream disruption of progenitor recruitment, osteoangiogenic coupling, and matrix deposition. Infection-associated inflammation, for example with Staphylococcus aureus, can be associated with fibrotic or disorganized repair tissue and impaired vascular integration.^218^ Sterile but persistent inflammation can similarly arrest repair and has been associated with impaired callus formation and atrophic phenotypes in experimental models.^10,150^ Persistent inflammation may therefore predispose to nonunions with limited regenerative potential. This highlights that biologic augmentation is unlikely to overcome unresolved local drivers such as infection, necrotic tissue, or dead space unless debridement, stability, soft-tissue management, and infection control are first addressed.

##### Open fracture/soft-tissue envelope damage

Severe soft-tissue injury, especially in open fractures, disrupts regional perfusion, hematoma stability, and early molecular gradients. It also causes tissue necrosis and increases contamination burden, collectively prolonging inflammation and reducing the effective osteogenic cell pool at the fracture margins.^219^ The periosteum and adjacent soft tissues are major sources of osteoprogenitors and early paracrine cues. Therefore, extensive stripping, crush injury, or devitalization can blunt periosteal callus formation and impair progression from granulation tissue to organized cartilaginous and osteogenic repair. This biases healing toward delayed union and biologically weak, often atrophic-appearing, nonunion in high-energy injury patterns.^220^ Open injuries also carry a substantially increased risk of fracture-related infection.^221,222^ Persistent bacterial burden, repeat debridement, periosteal stripping, and local ischemia act through overlapping mechanisms, including loss of progenitor niches, reduced oxygen and nutrient delivery, impaired endothelial ingrowth, and sustained inflammatory dysregulation. Together, these factors suppress osteogenesis and angiogenic progression, so that even with adequate fixation, bridging and callus maturation may remain limited.^219,223,224^ Severe soft-tissue compromise is therefore best understood as a combined disruption of progenitor supply, vascular support, and inflammatory control that predisposes to atrophic or infected nonunion unless the envelope and local contamination are adequately addressed.

##### Segmental bone loss/critical-sized defects

Segmental defects involve true loss of bone continuity and surface area, so healing fails not only from impaired biology but also from absence of the structural substrate needed for bridging. As defect length increases, the available osteogenic interface at the fracture ends becomes too small relative to the gap. Interfragmentary motion and strain depend on loading, fixation stiffness, and gap geometry and may remain mechanically unfavorable, while vascular disruption with limited angiogenic penetration impedes formation and maturation of a stable callus across the defect.^225^ Even when the fracture ends remain biologically active, the regenerate must traverse a gap that exceeds the capacity of spontaneous periosteal and endosteal repair. Union is therefore unlikely without restoration of both mechanical continuity, through stable load sharing, and biologic volume, through scaffold, graft, or vascularized tissue.^225–227^ Clinically, these cases are commonly managed with staged reconstructive strategies such as induced membrane techniques, distraction osteogenesis or bone transport, vascularized grafts, and/or large-volume grafting with fixation revision. Failure of fusion-like grafting or single adjunctive biologics in this setting often reflects indication mismatch rather than intrinsic inefficacy of the biologic. As segmental defects are reconstruction-dominant and often treated in staged fashion, they are often excluded from union-acceleration trials or analyzed separately, depending on trial design and indication.^226^ Critical-sized defects therefore represent one of the clearest examples of a substrate-loss constraint. Successful treatment depends on reconstructive strategies that restore span, vascularized tissue, and biologic volume rather than on incremental modulation of standard healing pathways.

#### Genetic and epigenetic modulators

Beyond systemic and local influences, genetic variation and epigenetic regulation modify individual susceptibility to impaired repair. Rather than producing a unique nonunion phenotype on their own, these factors shift the threshold at which inflammatory, osteogenic, angiogenic, and remodeling pathways fail under stress, consistent with a multifactorial susceptibility model.

##### Genetic factors

Genetic influences on fracture-healing outcomes have been reported in candidate-gene case–control studies and summarized in systematic reviews linking single nucleotide polymorphisms (SNPs) to aseptic long-bone nonunion. These associations particularly involve BMP-pathway genes such as *NOG*, *SMAD6*, and *BMP4*, as well as other repair-related genes including *FGFR1*, *NOS2*, *CYR61*, and *IL1B*.^228–230^ Conversely, polymorphisms in genes such as *MMP13*, *BMP6*, and *FAM5C* have been reported more often in uneventful union cohorts, suggesting possible protective associations.^228,230^ However, effect sizes are typically modest and limited by cohort size and replication. These limitations support a multifactorial or polygenic susceptibility model and motivate larger genome-wide association studies (GWAS).^228,231^ Currently identified variants are therefore best viewed as risk modifiers that bias repair toward particular mechanistic failure modes rather than as clinically actionable predictors of a distinct nonunion phenotype.

##### Epigenetic regulation

Epigenetic mechanisms, including DNA methylation, histone modifications, and noncoding RNAs, shape gene-expression programs during repair and influence osteogenic and chondrogenic differentiation as well as regeneration.^232^ Unlike inherited genetic variants, these mechanisms are dynamically modified by age, inflammation, metabolic disease, and local injury conditions, making them a plausible interface between risk exposure and biological healing response. Experimentally, chondrocyte DNMT3b loss delays endochondral ossification and fracture repair and impairs angiogenesis-related processes.^233^ Human nonunion tissue has been associated with altered noncoding RNA profiles, supporting the broader concept that epigenetic and post-transcriptional regulation may contribute to impaired osteogenic, vascular, and inflammatory repair programs.^232^ Because human fracture-healing epigenetic data remain limited, these alterations should be interpreted as emerging contributors to impaired repair rather than established clinical biomarkers. Collectively, epigenetic alterations intersect with the signaling modules discussed in Section 3 and appear to reshape the same immune, progenitor, vascular, endochondral, and remodeling failure modes rather than producing a single uniform nonunion pattern.

Taken together, the modulators in Sections “Systemic modulators”–”Genetic and epigenetic modulators” can be understood as upstream constraint setters that bias the fracture site toward mechanical failure, limited biologic capacity, persistent inflammation or infection, or substrate loss. Sections “Therapeutic strategies for fracture healing” and “Clinical translation and regulatory landscape” evaluate interventions through this same lens, asking whether a therapy corrects the dominant bottleneck rather than assuming that all delayed unions and nonunions are biologically equivalent.

## Therapeutic strategies for fracture healing

This section reviews therapeutic strategies for fracture healing (Table 2) in relation to the mechanical, biological, and substrate-loss constraints outlined in Section “Disruption of fracture healing: modulators and mechanisms”. It covers interventions used in acute fractures as well as in delayed union and nonunion, where revision fixation is often combined with biological augmentation.^150^ For each therapy class, the emphasis is on the principal mechanistic target, the strongest representative preclinical and clinical evidence, the main limitation to broader use, and the failure mode the intervention is most suited to address. The section is organized from mechanical stabilization and reconstructive strategies to biologic augmentation.

**Table 2:** Therapeutic strategies for fracture repair

Modality	Typical use/delivery	Main repair target	Main limitation
Fixation devices	Internal or external fixation; revision fixation when needed	Restores alignment and strain environment; corrects mechanical instability	Context-dependent construct mechanics; overly rigid or poorly matched constructs may limit repair and fixation does not by itself correct impaired biology
Physical adjuncts (PEMF, LIPUS)	Noninvasive external adjunct	Provides biophysical stimulation linked to mechanotransduction and callus signaling	Inconsistent clinical benefit; later larger LIPUS trials and systematic reviews show no meaningful improvement in healing or functional recovery
Advanced surgical interventions (Masquelet, bone transport/distraction)	Staged reconstruction or segmental transport	Addresses major substrate loss and restores bone continuity in defects	Technically demanding; high complication burden; evidence largely observational
Autograft	Local implantation, often with fixation revision	Supplies osteogenic cells, osteoinductive signals, and scaffold in one graft	Limited harvest volume and donor-site morbidity
Allograft/DBM/CBM	Structural graft, void filler, or extender depending on product	Expands biologic volume or structural support when autograft is insufficient	Marked product-to-product variability; incorporation and biologic activity less reliable than autograft
Synthetic bone substitutes	Calcium phosphate ceramics, calcium sulfate materials, bioactive glasses, calcium phosphate cements, or composites placed in contained defects	Provides osteoconductive scaffold and defect fill	Performance depends on containment, vascularity, and resorption match
Platelet concentrates (PRP/PRF)	Local injection or intraoperative adjunct	Augments early recruitment, angiogenesis, and matrix signaling	Poor standardization and heterogeneous clinical outcomes
Bone marrow aspirate concentrate (BMAC)	Percutaneous injection and/or intraoperative adjunct during revision surgery	Delivers autologous marrow cells and soluble mediators as biologic augmentation	Low and variable progenitor content; no validated potency threshold
Growth factors and cytokines (e.g., BMPs)	Local delivery with carrier or scaffold	Boosts progenitor recruitment, osteogenic differentiation, and osteoangiogenic coupling	Strong dependence on dose, timing, and carrier; adverse-effect risk with uncontrolled exposure
Pharmacologic agents	Systemic therapy	Supports anabolic or remodeling pathways	Systemic delivery mismatches a local, phase-specific process; evidence in nonunion is limited
Monoclonal antibodies	Systemic injection	Shifts remodeling pathways toward net anabolism or reduced resorption	High cost and uncertain fracture-site exposure; fracture-specific efficacy remains unproven
Cell-based therapies	Local cell delivery, usually with scaffold/carrier	Provides paracrine immunomodulatory, angiogenic, and osteogenic support	Product definition, manufacturing consistency, and cell retention remain major barriers
Tissue-engineered therapies	Implanted ex vivo construct or template	Reconstructs large defects or refractory nonunions with living tissue-like grafts	Vascularization, scale-up, and manufacturing complexity limit translation
EV-based therapies	Local EV delivery, often scaffold-assisted	Cell-free amplification of osteogenic, angiogenic, and immunoregulatory signaling	Poor standardization of EV yield, cargo, storage, and potency testing
Genetically modified cells	Locally implanted engineered cells	Provides sustained local production of regenerative factors	Vector safety, expression control, and GMP complexity
Gene- and RNA-based therapies	Local acellular genetic delivery	Reprograms host cells for local factor production in situ	Delivery efficiency, biodistribution, safety control, and product standardization

Summary of the therapeutic classes discussed in the updated main text, organized by typical use/delivery, principal repair target, and the main limitation affecting clinical effectiveness or translation. Modalities span mechanical optimization and reconstructive strategies through biologic augmentation, pharmacologic approaches, and advanced regenerative platforms. For investigational modalities, the listed use/delivery reflects the most commonly studied approach in preclinical and/or early clinical literature rather than an established clinical protocol

*BMAC* bone marrow aspirate concentrate, *BMP* bone morphogenetic protein, *CBM* cellular bone matrix, *DBM* demineralized bone matrix, *EV* extracellular vesicle, *GMP* good manufacturing practice, *LIPUS* low-intensity pulsed ultrasound, *PEMF* pulsed electromagnetic fields, *PRF* platelet-rich fibrin, *PRP* platelet-rich plasma

### Fixation devices

Fixation remains the primary intervention in both acute fracture care and revision surgery because successful healing first depends on restoration of an appropriate mechanical environment. Alignment, construct stability, and interfragmentary strain determine whether local loading supports callus formation or instead sustains shear, torsion, and instability that prevent bridging.^234,235^ Preclinical mechanobiology studies support the principle that constructs allowing controlled axial micromotion can enhance callus formation and mechanical restoration compared with overly rigid fixation.^236,237^ Clinically, plating, intramedullary nailing, and external fixation remain the foundation of long-bone fracture management, including open tibial shaft fractures and mechanically compromised nonunions.^234,238^ Comparative superiority of one construct over another is difficult to establish because outcomes are strongly confounded by fracture location and pattern, soft-tissue injury, reduction quality, comminution, weight-bearing protocol, and whether fixation is used in a primary or revision setting. The literature therefore supports matching construct mechanics to fracture configuration and biological context rather than assuming universal superiority of any single device.^234,235,237^ Dynamic and elastic fixation strategies, including dynamization and far-cortical locking, aim to preserve beneficial axial motion while maintaining overall stability.^237,239^ Their benefit appears context dependent and varies with fracture configuration, timing, and residual biological capacity. Likewise, antibacterial-coated implants or newer stiffness-modulating constructs may be useful in selected indications, but primarily when they address a defined infection-related or mechanical problem.^234,235,240^

### Physical adjuncts

Low-intensity pulsed ultrasound (LIPUS) and pulsed electromagnetic fields (PEMF) are noninvasive adjuncts used in acute fractures and impaired healing.^241,242^ Their rationale lies in partial overlap with endogenous mechanotransduction, as ultrasound and PEMF have each been proposed to influence ion-channel signaling, calcium influx, and downstream osteogenic programs linked to callus formation and endochondral repair.^243,244^ Preclinical studies support stimulus-dependent biological activity. LIPUS effects vary with treatment stage and duration,^245^ whereas PEMF enhances osteogenic and reparative responses in experimental osteotomy models and in vitro systems in an exposure-dependent manner.^243,246^ Clinical evidence is inconsistent and differs between modalities. For LIPUS, early trials and meta-analyses suggested possible reductions in radiographic or clinical healing time.^247^ However, later larger randomized evidence in surgically treated tibial fractures and subsequent systematic reviews found no meaningful improvement in healing or functional recovery.^248,249^ Evidence in established delayed union or nonunion remains more heterogeneous and less definitive.^250^ PEMF has shown exposure-dependent biological effects in preclinical and In vitro systems and has been used clinically in impaired healing, but clinical benefit remains dependent on indication, protocol, and patient selection.^243,246,251^ Interpretation is limited by substantial heterogeneity in stimulation protocols, treatment schedules, fracture types, and patient selection.^244,247^ These modalities are best regarded as noninvasive, low procedural-burden, selective adjuncts with uncertain and indication-dependent effect sizes rather than standard union-accelerating therapies or stand-alone treatments. Their most plausible role is in selected, mechanically stable fractures in which modest enhancement of mechanotransductive signaling may assist repair. They should not be expected to compensate for mechanical instability or major biological failure.^245,247,250,251^

### Advanced surgical interventions

Advanced surgical reconstruction is used mainly for segmental bone defects, complex nonunions, and limb-salvage settings in which standard fixation and biologic augmentation alone are unlikely to restore continuity. In these situations, the goal is not simply to accelerate repair, but to recreate a mechanically stable and biologically permissive environment for regeneration across a large defect or chronically failed fracture site. The Masquelet technique is most applicable when healing is limited by segmental substrate loss and the need for staged reconstruction. Its main promise lies in converting an otherwise poorly regenerative defect into a graft-receptive chamber with revascularization potential and local osteogenic and angiogenic support.^252,253^ Reported union rates can be favorable, with one systematic review and meta-analysis estimating union in approximately 89.7% of cases, although complication rates remain substantial.^254^ Outcomes depend heavily on defect size, infection control, fixation quality, soft-tissue coverage, and graft biology rather than on membrane formation alone.^253–255^ Distraction osteogenesis is most useful when reconstruction must address not only nonunion or bone loss, but also segmental shortening, deformity, or compromised soft tissue. Its main advantage is that it generates living vascularized bone while restoring length and alignment, making it particularly valuable when graft-based reconstruction is limited. Clinical effectiveness is well documented, but treatment burden is high, and complications frequently occur at the docking site, including delayed union and the need for secondary procedures.^256,257^ These approaches are therefore best viewed as reconstructive platforms rather than biologic augments. Their promise lies in enabling healing where defects or nonunions are too large or structurally complex for simpler strategies, but success depends primarily on indication selection, infection control, fixation, soft-tissue management, and restoration of a biologically permissive reconstruction environment rather than on the intrinsic potency of any single biologic adjunct.

### Autologous and allogeneic bone grafts

Autologous and allogeneic grafts are used when healing is limited by substrate loss, cavitary defects, or insufficient biological volume. Autograft remains the clinical reference standard because it combines osteogenic, osteoinductive, and osteoconductive properties in a single material.^258,259^ Allograft is the main alternative, but performance depends strongly on graft type and processing. Mineralized structural allografts primarily provide mechanical support and osteoconduction, whereas demineralized bone matrix is used mainly as an osteoinductive or osteoconductive extender rather than as a structural graft.^260–262^ Because much of the clinical evidence for demineralized bone matrix comes from adjacent settings such as spinal fusion and other orthopedic reconstruction contexts such as hindfoot arthrodesis or osteotomy, direct long-bone fracture or nonunion efficacy should not be assumed from this literature alone.^261–264^ Cellular allografts aim to add viable cells, whereas decellularized scaffolds aim to preserve matrix-derived biological cues; however, most clinical evidence for these products remains outside long-bone delayed-union and nonunion care, and equivalence to autograft cannot be assumed.^265–267^ These materials are best suited to contained substrate-loss problems in mechanically competent environments. Autograft remains the most reliable biological option, whereas processed allografts expand reconstructive possibilities when greater volume or structural support is required, albeit with more variable incorporation and biological performance.

### Synthetic bone substitutes

Synthetic bone substitutes, including calcium phosphate ceramics, calcium sulfate materials, bioactive glasses, calcium phosphate cements and other cement formulations, and composites, are used mainly to address contained substrate-loss problems rather than intrinsic biologic failure.^268,269^ Their main advantage is provision of a reproducible osteoconductive scaffold without donor-site morbidity, and some formulations also offer injectability, structural void filling, or can be formulated for local antibiotic delivery. Performance depends strongly on material architecture, as porosity and surface characteristics influence cell infiltration, vascular ingrowth, and remodeling.^270–272^ Clinically, these materials are most useful in well-contained, mechanically stable defects, such as metaphyseal voids after fracture reduction or cavitary bone loss encountered during revision. Their role is primarily scaffold-based, since most lack viable osteogenic cells and have limited intrinsic osteoinductive activity. Outcomes therefore depend as much on defect containment, vascularity, and fixation quality as on the material itself, and comparison across studies is complicated by major heterogeneity in indications, formulations, resorption kinetics, and outcome measures.^268,269^ Their main limitation is resorption mismatch. If resorption is too rapid, structural support is lost before sufficient bone forms; if too slow, remodeling and incorporation are delayed.^269^ Synthetic substitutes therefore perform best as osteoconductive components of a broader reconstructive strategy in contained, well-vascularized defects.

### Platelet-based therapies and concentrates

Platelet-rich plasma (PRP) is an autologous platelet concentrate investigated and sometimes used as an adjunct during fracture fixation and revision surgery. Its rationale is early biologic amplification: PRP releases mediators such as PDGF, TGF-β, VEGF, and IGF-1, which may support progenitor recruitment, angiogenesis, and early osteogenic signaling. Experimental evidence supports biological activity in selected models, including effects on vascular response, callus formation, and structural healing, but these effects depend strongly on preparation method and delivery context.^273–275^ Clinical evidence is less convincing than the preclinical rationale. In long-bone delayed union and nonunion, systematic-review evidence suggests promising union rates in some studies but remains limited by small study numbers, few randomized controlled trials, heterogeneous patient populations, and variable treatment protocols.^241^ A central problem is product heterogeneity: preparations labeled as PRP differ in platelet concentration, leukocyte content, activation method, growth-factor release, and inflammatory profile, making comparison difficult and limiting reproducibility.^276^ PRP is therefore best regarded as a low-risk autologous adjunct with uncertain effect size rather than a defined biologic therapy. Its most plausible role is in mechanically stable settings requiring modest early biologic support, but meaningful clinical use will depend on much better standardization of product composition, delivery, and retention at the fracture site.

### Bone marrow aspirate concentrate

Bone marrow aspirate concentrate (BMAC) is a point-of-care autologous orthobiologic used to augment fracture repair by delivering a heterogeneous mixture of marrow-derived cells and soluble mediators.^277,278^ Although often described as a stem-cell therapy, MSC content is low, variable, and inconsistently enriched across patients and preparation systems.^277^ Preclinical studies generally support biological activity, including increased bone formation and, in some models, improved mechanical outcomes.^279^ However, interpretation is limited by variability in harvest site, concentration method, and host condition, and it remains unclear which cellular or soluble components account for any observed effect. Clinically, BMAC is used either by percutaneous injection into mechanically stable nonunions or as an adjunct during revision surgery with fixation optimization and/or grafting.^280^ Outcomes are often encouraging, but the evidence base is dominated by observational studies and remains highly heterogeneous with respect to nonunion phenotype, infection status, defect size, patient risk factors, preparation method, and delivered cell dose.^277,280^ Although some reports suggest a dose–response relationship, this has not yet translated into validated potency thresholds for clinical use.^278,280^ BMAC is best regarded as a biologic-capacity adjunct rather than a defined cell therapy. Its most plausible role is in mechanically stable environments requiring augmentation of local biologic activity, but its clinical value is limited by poor product definition, which restricts reproducibility, potency assessment, and comparative evaluation.^280^

### Growth factors and cytokines

Growth factors and cytokines target biological bottlenecks such as impaired progenitor recruitment, osteogenic differentiation, and osteoangiogenic coupling. In preclinical models, local delivery of factors such as FGF-2,^281^ VEGF,^282,283^ and PDGF-BB^284^ can increase callus formation, mineralization, angiogenesis or mechanical strength, demonstrating that targeted signal augmentation can influence repair. However, these data do not imply that single-factor therapy is sufficient across the heterogeneity of delayed union, nonunion, and segmental-defect phenotypes. Among these agents, recombinant human BMP-2 (rhBMP-2) has the strongest translational record. Large-animal studies and selected human trials support its ability to enhance bone formation and reduce secondary interventions in defined clinical settings.^285,286^ In the pivotal BESTT trial, adjunctive rhBMP-2 reduced secondary interventions and accelerated fracture and wound healing in open tibial fractures relative to standard care.^285^ However, later randomized evidence in open tibial fractures treated with reamed nailing did not show clear acceleration of healing, indicating that clinical benefit depends on indication, fixation context, dose, and delivery format.^287^ This remains one of the clearest examples that local growth-factor delivery can improve clinically relevant outcomes in fracture repair, but also illustrates the context dependence of this approach. At the same time, rhBMP-2 illustrates the limits of this strategy. Efficacy depends strongly on dose, timing, carrier, and spatial confinement. High dose, poorly confined release, or off-label use, especially in anatomically constrained settings, can increase the risk of ectopic ossification, local inflammatory complications, and other adverse events.^288^ More broadly, growth-factor therapy is constrained by short protein half-life and difficult local pharmacokinetics, making the carrier a determinant of efficacy and toxicity rather than a passive vehicle.^289,290^ Empirical combination of factors is not automatically beneficial because pathway crosstalk, lineage stage, and temporal sequence strongly influence outcome.^284,291–294^ Local growth-factor therapy is therefore best viewed as a potent but narrow biologic amplifier. It is most plausible when local biologic insufficiency is the dominant problem in a well-stabilized site; safety and delivery constraints, including dose, carrier, and spatial-control requirements, limit broader routine use.

### Pharmacological agents

Pharmacological agents can influence fracture repair by modulating pathways involved in osteogenesis, remodeling, angiogenesis, and inflammation. In preclinical models, pathway-modulating agents such as Smoothened agonists, statins, prostaglandin analogs, and PTH1R agonists have improved bone-regenerative readouts, including callus formation, mineralization, or mechanical strength, depending on the model.^295–299^ However, most are approved for other indications or remain experimental rather than being developed specifically as fracture-healing drugs, and human fracture-healing evidence remains limited. Among these agents, teriparatide is the best-studied clinical example. In a randomized double-blind trial of postmenopausal women with distal radial fractures, teriparatide 20 μg/day shortened time to cortical continuity compared with placebo, whereas the higher dose did not provide additional benefit.^300^ Subsequent fracture-healing reviews, off-label reports, and delayed-union/nonunion case series suggest at most modest and heterogeneous effects on healing time, while evidence in delayed union and nonunion remains limited to small case series or low-level clinical evidence.^301–305^ Teriparatide therefore serves mainly as proof of concept for anabolic pharmacologic support rather than as an established fracture-healing therapy. A central limitation is delivery mismatch. Fracture healing is local and phase dependent, whereas most pharmacological agents are delivered systemically, making fracture-site exposure difficult to control and increasing the risk of off-target effects or mistimed pathway modulation.^306,307^ These agents are best considered adjuncts for reduced anabolic drive rather than treatments for instability, infection, soft-tissue compromise, or major substrate loss.

### Monoclonal antibodies

Monoclonal antibodies target extracellular regulators of bone remodeling, particularly pathways controlling osteoblast and osteoclast activity.^48^ In preclinical fracture models, inhibition of Wnt antagonists such as sclerostin, and in some models DKK-1, increases Wnt/β-catenin signaling, enlarges callus, and improves mechanical strength.^308–310^ However, this mechanistic promise has not translated into convincing fracture-specific clinical benefit. For example, Romosozumab is approved for fracture-risk reduction in osteoporosis, but it is not licensed to accelerate acute fracture healing or treat delayed union or nonunion.^311,312^ Large osteoporosis trials were not designed to assess healing of existing fractures, and a dedicated phase II hip-fracture study of romosozumab showed no significant improvement in radiographic or clinical healing outcomes compared with placebo.^313^ Safety data around fracture occurrence are reassuring, but they do not establish efficacy for fracture repair.^314^ As with other systemic therapies, the main limitation is delivery mismatch. Fracture healing is spatially and temporally restricted, whereas current antibody therapy is systemic, expensive, parenteral, and may also be limited by poor penetration into poorly vascularized repair tissue.^315–317^ Rare but important adverse effects, particularly medication-related osteonecrosis of the jaw with antiresorptive agents such as denosumab, further narrow the risk–benefit margin in selected patients.^318–321^ Monoclonal antibodies are therefore best regarded as systemic modifiers of bone biology rather than currently established fracture-healing therapies. What is lacking is not further mechanistic rationale, but fracture-specific comparative evidence.

### Cell-based therapies

Cell-based therapies aim to improve fracture repair by delivering viable cells, including MSCs and other progenitor populations, to enhance local paracrine support and biologic activity rather than to generate mature tissue constructs directly.^322,323^ In preclinical fracture models, MSC delivery often increases callus formation, vascularization, and mechanical strength, with most studies attributing benefit to secreted trophic and immunomodulatory signals rather than durable engraftment.^322,324^ These effects have been linked to pathways regulating osteogenesis and angiogenesis, including Wnt/β-catenin, BMP/Smad, and hypoxia-associated signaling.^11,322^ Clinical translation remains limited. Most human studies involve long-bone delayed union or nonunion and consist of small series or early-phase studies with heterogeneous cell-processing protocols, delivery methods, and endpoints.^325–327^ Marcacci et al. reported healing within 6 months in four patients with diaphyseal defects treated with autologous bone marrow-derived osteoprogenitors on hydroxyapatite scaffolds,^328^ and Kim et al. reported significantly greater early radiographic callus formation after treatment with autologous cultured osteoblasts than with observation alone in a multicenter randomized trial.^329^ These findings support feasibility but do not establish superiority over contemporary revision surgery with grafting and optimized fixation. CD34⁺ progenitor cell approaches represent a distinct strategy and have likewise been reported to show faster radiographic healing than historical controls, but remain limited by small sample size, reliance on historical comparison, and incomplete standardization of dose and potency criteria.^323^ The main translational barrier is product definition. Autologous approaches reduce immunogenicity, but donor variability, delayed availability, and inconsistent culture expansion limit reproducibility. Allogeneic products offer greater scalability, but require tighter control of immunogenicity and manufacturing consistency.^322^ More advanced options such as iPSC-derived MSC-like cells remain preclinical because cost and safety remain unresolved.^330,331^ Systemic administration is also inefficient, as fracture-site homing is poor and intravenously delivered MSCs are subject to pulmonary first-pass sequestration, making local delivery the more plausible route for most fracture indications.^322,326^ Cell-based therapies are best viewed as biologic-support strategies with strong preclinical rationale but insufficient comparative clinical evidence. Progress depends on standardized products, validated potency assays predictive of clinical efficacy, and trials showing benefit over established surgical care.

### Tissue-engineered therapies

Tissue-engineered therapies extend cell-based approaches by implanting ex vivo-generated cell–material constructs and are particularly attractive for critical-sized bone defects and biologically compromised or refractory nonunions, where both structural support and regenerative cues are needed. A major challenge is not only osteogenic differentiation, but also maintenance of cell survival and function at clinically relevant scale. As construct size increases, oxygen and nutrient diffusion become limiting, making early vascular integration a key determinant of post-implantation viability and remodeling.^332^ Current preclinical strategies therefore aim to improve construct integration by combining osteogenic, angiogenic, and endochondral design principles, although these approaches may increase manufacturing complexity and translational burden. Endochondral approaches exploit the relative hypoxia tolerance of cartilage templates, which subsequently remodel into bone in vivo, and have shown efficacy in critical-sized defect models.^333,334^ Acellular or callus-mimetic constructs suggest that off-the-shelf approaches may be feasible, although reproducibility and long-term functional equivalence remain unresolved.^335,336^ Clinical translation remains early. For example, in a prospective multicenter phase II trial, adipose-derived mesenchymal stromal cells combined with tricalcium phosphate achieved healing in 10 of 12 patients with atrophic long-bone nonunion, supporting feasibility but not comparative efficacy.^327,337^ A major translational obstacle is achieving reliable vascular integration and consistent manufacture at clinically relevant scale. These therapies are likely to require standardized potency testing, scalable good manufacturing practice (GMP) production, and reliable in vivo survival after implantation.^332,337^ Additional challenges include vascular integration and, for advanced cell sources such as iPSC-derived progenitors, safety and regulatory complexity.^331,338^

### EV-based therapies

EVs, including exosomes and microvesicles, are being developed as cell-free mediators of bone repair by transferring proteins, lipids, messenger RNAs (mRNAs), and miRNAs that regulate osteogenesis, angiogenesis, and inflammation. Their therapeutic rationale is that they amplify a native signaling system: EVs released by MSCs, osteoblasts, ECs, and immune cells already modulate pathways such as BMP/Smad, Wnt/β-catenin, and HIF–VEGF during fracture repair. Preclinical evidence is substantial but remains largely mechanistic. MSC-derived EVs have repeatedly enhanced fracture or bone-defect repair in preclinical models through paracrine signaling, with reported effects on osteogenesis, angiogenesis, and inflammatory resolution.^339–341^ For example, EV-associated miR-335 has been shown to promote osteogenesis and callus formation through activation of Wnt/β-catenin signaling in murine fracture models.^342^ Other studies show that EV cargo such as miR-29b-3p can enhance neovascularization through PTEN suppression and PI3K/Akt activation,^343^ while miR-136-5p promotes osteoblast proliferation and differentiation through LRP4 inhibition and Wnt/β-catenin activation.^344^ Local delivery using hydrogels, scaffolds, or injectable depots improves retention and regenerative effect, indicating that EV efficacy depends as much on delivery format as on cargo selection.^345,346^ The main translational barrier is standardization of the therapeutic entity itself. EV yield is often low, cargo composition varies with cell source and culture conditions, and there is no universally accepted workflow for isolation, storage, characterization, or potency testing. This heterogeneity complicates reproducibility, dose definition, and regulatory classification.^339,347,348^ EV-based therapies are therefore best viewed as mechanistically sophisticated but still preclinical signal-delivery platforms. Their appeal lies in being cell-free and pathway-rich; their limitation is that neither dose nor potency is yet defined well enough to support routine clinical translation.

### Genetically modified cells

Genetically modified cell therapies aim to improve fracture repair by engineering MSCs or related progenitors to provide sustained local production of osteogenic, angiogenic, or immunomodulatory factors.^349^ In principle, this approach addresses a central limitation of unmodified cell therapy: low and variable endogenous signaling output. RUNX2-overexpressing MSCs have shown osteogenic repair effects in an adjacent osteonecrosis-related bone-repair model,^350^ while BMP-2-transduced MSCs and related engineered MSC strategies have been investigated more directly in nonunion and defect models.^351,352^ These effects depend on local implantation within scaffolds, hydrogels, or ECM-based carriers that retain the cells and concentrate paracrine output. Mechanistically, efficacy is mediated through BMP/Smad, Wnt/β-catenin, and VEGF/HIF-driven programs, but these same pathways also create the risk of overshooting. Spatiotemporal control of transgene expression is therefore essential, as unregulated expression can cause ectopic ossification, aberrant angiogenesis, or other off-target tissue effects.^353,354^ The main challenge is clinical deployability. Translation is constrained by vector-related safety issues, including insertional mutagenesis, off-target transduction, and immune activation, together with variable gene-transfer efficiency and the complexity and cost of large-scale good manufacturing practice (GMP) manufacturing.^355–357^ These are core development barriers rather than secondary technical issues. Genetically modified cells therefore remain experimental intensifications of cell therapy rather than near-term clinical options for fracture repair. They are most defensible in severe defect or salvage settings where simpler cell-based or graft-based approaches are inadequate, but they currently lack the safety, manufacturing simplicity, and regulatory tractability needed for routine use.

### Gene and RNA-based therapies

Gene- and RNA-based therapies aim to reprogram cells at the fracture site by delivering acellular genetic payloads that induce local production of osteogenic, angiogenic, or immunoregulatory mediators.^354,358^ This strategy addresses a key limitation of recombinant protein therapy by replacing repeated supraphysiologic dosing with localized, endogenous factor production. Platforms include plasmid DNA, viral vectors, synthetic mRNA, RNA interference, miRNA delivery, and, more experimentally, CRISPR/Cas-based editing systems.^354,359,360^ Preclinical studies show that local delivery of genes encoding osteogenic or angiogenic factors, including RUNX2^361^ or VEGF,^362^ can enhance mineralized tissue formation, vascularization, and repair in defect and nonunion models. RNA-based approaches have also shown bone-regenerative activity. For example, scaffold-mediated miR-26a delivery promotes osteogenic differentiation and angiogenic–osteogenic coupling.^359,363^ CRISPR/Cas strategies remain at an early proof-of-concept stage for skeletal repair.^360^ Clinical translation remains limited. Gene-activated matrices are among the more advanced examples, with early clinical reports evaluating localized plasmid-delivering scaffolds, including VEGFA plasmid-based bone substitutes, in restricted bone reconstruction indications.^364,365^ However, these studies remain small and indication-specific and do not yet establish routine applicability to long-bone fracture repair. The main barriers are platform-specific but converge on delivery control. Non-viral DNA delivery is often inefficient and transient, whereas viral vectors raise concerns about insertional mutagenesis, off-target transduction, and immune activation.^354,366^ RNA therapeutics avoid genomic integration but remain limited by instability, innate immune sensing, and carrier dependence.^359^ Across platforms, tissue targeting, biodistribution, regulated expression, reproducible manufacturing, and fracture-specific clinical standardization remain unresolved. Gene- and RNA-based therapies are therefore mechanistically powerful but developmentally demanding strategies. Their progress will depend less on identifying additional osteogenic targets than on solving delivery, safety, biodistribution, and manufacturing constraints compatible with fracture-specific clinical use.

Across these modalities, clinical translation depends not only on biologic efficacy but also on product definition, manufacturability, and evidence quality. These cross-cutting development constraints are addressed in Section 6.

## Clinical translation and regulatory landscape

Translating fracture-healing therapies is especially challenging because both the products and the clinical indications are heterogeneous. Candidate interventions span medicinal products, biologics, devices, cell-based constructs, engineered scaffolds, tissue-derived products, and combination products, while the clinical problems they aim to address range from acute fracture repair to delayed union, nonunion, segmental bone loss, infected defects, and fracture-related infection.^367,368^ These indications differ in biological mechanism, surgical context, comparator standard, clinical endpoint, and evidentiary requirements. Fracture-healing products therefore face translational and regulatory challenges at two interconnected levels: how the product is classified, based on intended purpose, principal mode of action, manipulation, origin, and components; and how the intended use is framed, for example as union acceleration, defect reconstruction, infection control, biological augmentation, or revision of failed repair. The discussion below focuses primarily on the European Union (EU) framework, with comparison to other major regulatory systems where relevant, and examines product classification, development pathways, the current clinical landscape, manufacturing and scalability, safety oversight, and HTA and reimbursement.

### Product Classification and regulatory pathways

In Europe, regulatory classification is primarily determined by a product’s intended purpose and principal mode of action (PMOA). Products acting principally through pharmacological, immunological, or metabolic mechanisms are regulated as medicinal products under Directive 2001/83/EC,^369^ whereas gene therapy medicinal products, somatic-cell therapy medicinal products, tissue-engineered products, and combined Advanced Therapy Medicinal Products (ATMPs) fall under the ATMP Regulation (EC) No 1394/2007.^370^ Human tissues and cells that do not meet ATMP criteria are generally governed by the tissues-and-cells framework under Directive 2004/23/EC,^371^ which will be replaced by the Substances of Human Origin (SoHO) Regulation (EU) 2024/1938 from 7 August 2027.^372^

Classification is often uncertain in fracture healing because many interventions combine scaffolds, devices, biologics, cell therapies, tissue handling, and intraoperative processing systems. This is particularly relevant in delayed union and nonunion surgery, where a single revision procedure may involve fixation, autologous tissue handling, graft substitutes, preparation systems, and local biologic augmentation. Autologous materials removed and reimplanted during the same procedure may, depending on the degree of manipulation, intended use, and national regulatory context, fall outside full medicinal-product pathways or be governed primarily through tissue/cell, hospital, or procedural oversight frameworks.^367,373^ By contrast, products whose principal intended action is physical or mechanical, and not achieved by pharmacological, immunological, or metabolic means, are regulated as medical devices under Regulation (EU) 2017/745, also known as the Medical Device Regulation (MDR). Many orthopedic implants and bone-regeneration devices fall into Class IIb or III, depending on intended purpose, duration of use, implantability, resorption, and incorporation of medicinal or biological components, and require Notified Body assessment before Conformité Européenne (CE) marking.^373–375^ In the United States, a broadly similar distinction applies: high-risk devices typically require Premarket Approval (PMA), whereas many moderate-risk devices proceed through the 510(k) pathway on the basis of substantial equivalence, and novel devices without a legally marketed predicate may use the De Novo pathway when general and/or special controls can provide reasonable assurance of safety and effectiveness.^376,377^

**Fig. 7:**
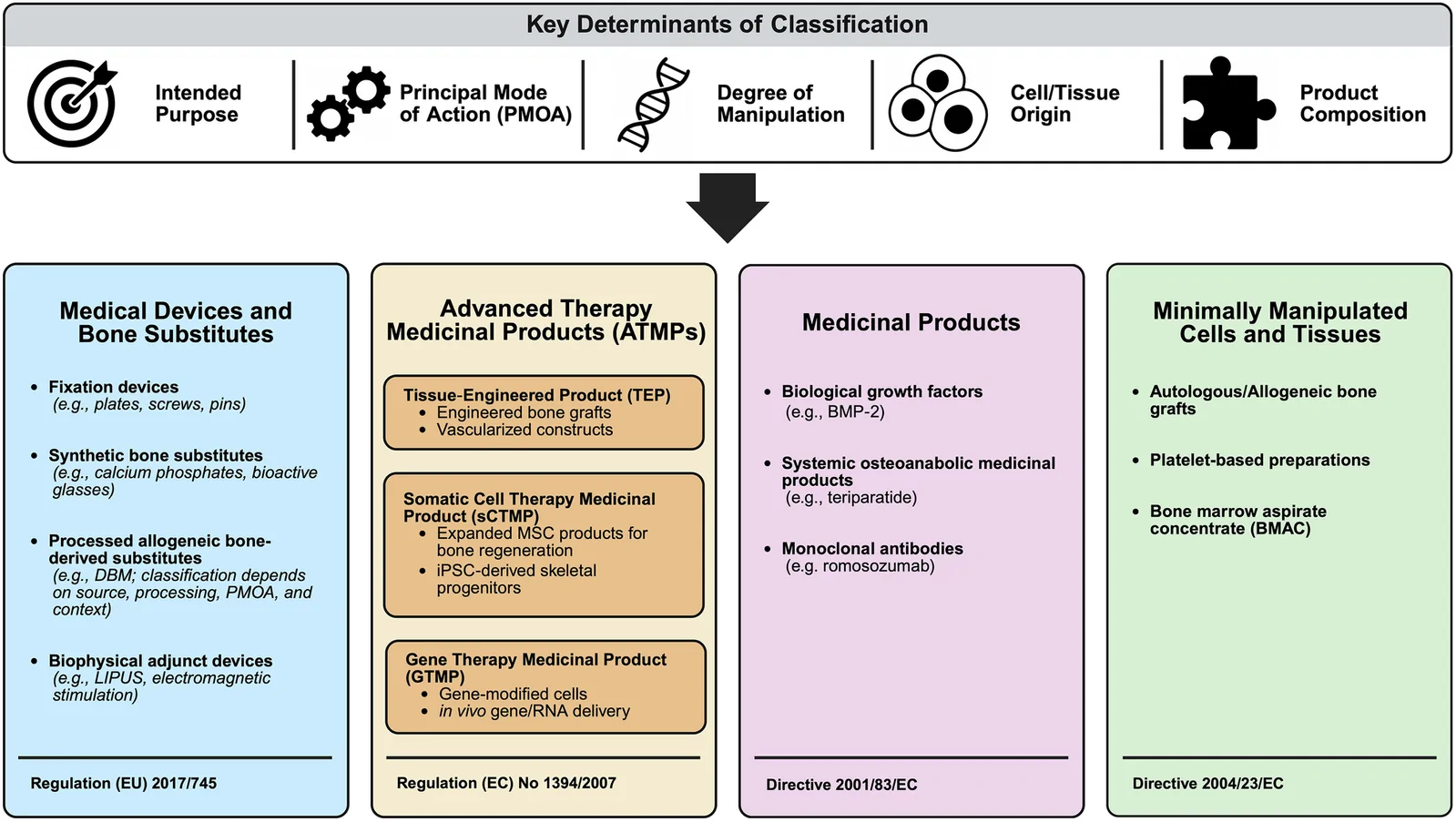
EU regulatory classification of fracture-healing therapies. Regulatory classification in the European Union is shaped by intended purpose, principal mode of action (PMOA), degree of manipulation, whether cells or tissues are intended to perform the same or a different essential function in the recipient than in the donor, cell/tissue origin, and product composition. These determinants influence whether fracture-healing products are regulated as medical devices or bone substitutes under Regulation (EU) 2017/745, Advanced Therapy Medicinal Products (ATMPs) under Regulation (EC) No 1394/2007, medicinal products under Directive 2001/83/EC, or under applicable human-tissue, blood, procedural, hospital, or national frameworks, including Directive 2004/23/EC where relevant. The categories and examples shown are schematic rather than class-wide legal assignments; classification is product-, process-, intended-use-, and Member-State-specific. Examples relevant to fracture and nonunion care include fixation devices, synthetic or allogeneic bone-derived substitutes, biophysical adjunct devices, tissue-engineered, somatic cell, and gene therapy medicinal products, biological growth factors, osteoanabolic medicinal products, monoclonal antibodies, bone grafts, platelet-based preparations, and bone marrow aspirate concentrate. Platelet-based preparations and bone marrow aspirate concentrate may be governed under different blood, tissue/cell, procedural, or hospital frameworks. Processed allogeneic bone-derived substitutes may fall into different categories depending on source, processing, intended use, PMOA, viability, and national context; human tissues and cells supplied under tissue frameworks should be distinguished from eligible non-viable or rendered-non-viable human-tissue derivatives that may fall under the Medical Device Regulation. This framework illustrates how regulatory classification can affect clinical translation, manufacturing requirements, evidence generation, and reimbursement. Created with BioRender.com

For FDA-regulated combination products, assignment is based on the primary mode of action, with the constituent part providing the principal therapeutic effect determining the lead regulatory route. Similar borderline questions arise in the EU through MDR, medicinal-product, ATMP, and tissue/cell rules, with the applicable route depending on intended purpose, principal mechanism, manipulation, and ancillary components.^373,374,376^ In fracture healing, this is not merely a legal technicality. Early misclassification can later force changes in manufacturing, preclinical testing, trial design, and commercial positioning, particularly for borderline products such as cell-seeded scaffolds, intraoperatively prepared orthobiologics, BMP-carrier constructs, antibiotic-eluting bone substitutes, and SoHO-derived bone products.^367,373,374,376^ Fig. 7 summarizes the key determinants of classification and the main EU regulatory categories relevant to fracture and nonunion care.

Although the EU framework provides harmonized legal categories, practical oversight involves several actors and routes. The European Medicines Agency (EMA) is central to ATMP authorization, tissue- and cell-based products that do not meet ATMP criteria remain linked to tissue/cell and national competent-authority frameworks, and medical devices are assessed through MDR conformity assessment by designated Notified Bodies.^371,374,375,378,379^ As a result, procedural requirements and timelines may differ even within a shared legal framework.

Outside Europe, major regulators such as the FDA, Japan’s Pharmaceuticals and Medical Devices Agency (PMDA), and China’s National Medical Products Administration (NMPA) also distinguish between medicinal-product, device, biologic, and regenerative-medicine pathways, although the precise categories and evidence requirements differ by jurisdiction.^380^ In fracture-healing indications, classification is especially consequential because it shapes the approval route, manufacturing strategy, comparator selection, and evidence expected for acute fracture repair, nonunion revision, and defect reconstruction. Early alignment between classification, manufacturing strategy, and clinical-development design is therefore essential.

### Development pathways and trial design

Clinical development of fracture-healing therapies broadly follows the standard regulatory pathways for medicinal products, ATMPs, and devices, but trial design is unusually challenging because fracture-healing failure is both biologically and mechanically heterogeneous.^381,382^ A therapy may target impaired osteogenesis, vascularization, or progenitor recruitment, yet be tested in patients whose dominant constraint is instability, infection, or major substrate loss. In this field, trial failure may therefore reflect poor alignment between mechanism and indication as much as lack of therapeutic activity.^367,368,382^

For medicinal products and ATMPs, development generally follows staged clinical evaluation, with early studies addressing safety, dose, feasibility, and biological activity, followed by exploratory efficacy and where appropriate, confirmatory comparison against standard care.^379,383,384^ For devices, including stimulation technologies and regenerative implants, development follows device-specific regulatory routes and generally requires performance, safety, and clinical evidence proportionate to device risk and intended use.^377^ In fracture healing, however, the key challenge is often not the formal pathway but the definition of the intended indication and endpoint. Acute fracture repair, delayed union, nonunion, segmental defect reconstruction, and fracture-related infection are often grouped under a single regenerative-development umbrella, even though they represent distinct clinical problems with different comparators, different risk profiles, and different dominant constraints on repair. Clinical programs should therefore specify whether the intervention is intended to accelerate expected union, rescue biologically impaired repair, reconstruct missing bone, control infection, or support revision surgery.

Endpoint selection is therefore a central challenge. Time to union, union rate, and reoperation remain the dominant outcomes, but definitions vary across studies and radiographic union does not always correspond to mechanical competence or patient recovery.^381^ Accordingly, fracture-healing trials should combine prespecified structural endpoints with clinically meaningful outcomes such as unrestricted weight-bearing, return to work or usual activity, revision surgery, pain, quality of life, and validated functional recovery.^381,382,384,385^ Where possible, radiographic endpoints should be prespecified using standardized criteria and interpreted alongside clinically meaningful outcomes such as time to unrestricted weight-bearing, return to work or usual activity, revision surgery, pain, and validated functional recovery. Independent adjudication committees and blinded radiographic review help reduce assessor bias, while comparators often reflect optimized fixation with or without graft augmentation.^381,382^

Trials should therefore stratify or exclude subgroups with clearly different dominant failure mechanisms, such as infected nonunion, large segmental defects requiring reconstruction, or overtly unstable constructs.^386–388^ This constraint-based approach is essential because union acceleration, defect reconstruction, and infection control are not interchangeable indications.^389^ Quantitative imaging and standardized radiographic scoring systems may eventually support more selective recruitment and interim refinement of study populations, and artificial intelligence (AI)-supported risk models remain an early-stage possibility; however, these approaches require fracture-specific validation before they can be used for trial enrichment or adaptive decision-making.^390–392^ Bayesian adaptive designs may also help address rare and heterogeneous nonunion populations when prospectively justified, adequately controlled, and prespecified in the trial protocol.^393,394^

Long-term follow-up remains necessary for implants, biologics, cell- and gene-based therapies, and regenerative devices, because delayed complications such as recurrent nonunion, osteolysis, implant failure, ectopic tissue responses, persistent inflammatory reactions, or late safety signals may not be captured by short-term radiographic analyses.^379,382,395^ Regulators and HTA bodies increasingly expect trial populations, subgroup analyses, and outcomes to reflect real-world clinical relevance as well as statistical rigor.^385,396,397^ In fracture-healing indications, radiographic bridging alone is therefore rarely sufficient; successful translation requires evidence that an intervention improves clinically meaningful outcomes for patients and supports decision-making by surgeons, payers, and health systems.^381,382^

### Current clinical landscape

The current clinical landscape of fracture-healing therapy remains mechanically anchored: stable fixation is still the indispensable foundation of repair, while grafting and graft substitutes remain the most established biological adjuncts for bone loss, delayed union, nonunion, and defect reconstruction.^268,389^ The field has expanded to include demineralized bone matrix (DBM), bioceramics, BMP-based products, marrow- and platelet-derived orthobiologics, and biophysical stimulation modalities such as electrical stimulation and LIPUS. In practice, most fracture and nonunion care still relies on fixation combined with autografts, allografts, DBM, synthetic substitutes, and selected stimulation technologies. Comparatively few adjuncts have fracture-healing- or nonunion-specific indications supported by substantial comparative evidence; many are instead used for void filling, structural support, fusion, infection-related reconstruction, or broader orthobiologic purposes according to product-specific claims, local practice, or off-label clinical judgment.^268,367,398,399^ Advanced cell-derived constructs, tissue-engineered grafts, and other next-generation biologics remain substantially less mature clinically and have not yet reached routine adoption outside selected investigational programs or highly specialized clinical settings.^269,368^

#### Established therapies and labeled adjuncts

Autologous bone grafting remains the clinical gold standard for fracture and nonunion repair.^258^ In Europe and the United States, conventional intraoperative autografts used within the same surgical procedure are generally managed under surgical practice, hospital governance, and relevant tissue-handling frameworks rather than formal marketing-authorization pathways.^367,400^ Allogeneic bone grafts, including cancellous chips, corticocancellous or structural blocks, and cortical strut allografts, are also routinely used for acute fractures with metaphyseal voids and for delayed union or nonunion reconstruction when osteoconduction and, in structural forms, mechanical support are required. In Europe, these products are typically supplied as human tissues through authorized tissue establishments under the current tissues-and-cells framework, transitioning to the SoHO framework from 7 August 2027, and are governed by tissue rules and local hospital oversight rather than a dedicated fracture-healing drug claim.^401^

Beyond grafting, only a limited number of exogenous biologic therapies have achieved fracture-healing or nonunion-related clinical translation. Recombinant BMP-2 is the best-studied example. Dibotermin alfa/rhBMP-2 is best described as an indication-specific adjunct rather than a broadly approved fracture-healing therapy, with selected approved indications in Europe and the United States including spinal fusion and acute tibial-fracture surgery.^367,400^ Randomized tibial-fracture evidence showed reduced treatment failure versus standard care, providing proof of principle that local biologic augmentation can improve clinically relevant outcomes.^285^ However, postmarket surveillance and broader safety literature identified important adverse effects, including ectopic bone formation and inflammatory complications, particularly in off-label settings.^288,402^ Earlier BMP-7/OP-1 experience showed promise in tibial nonunion, but this platform is now best discussed as historical clinical experience rather than a current routine fracture-healing option.^367,400,403,404^

Biophysical stimulation devices, including PEMF and LIPUS systems, have also been used as non-invasive adjuncts for fracture healing, delayed union, and nonunion. Some devices have carried fracture-related or nonunion-related indications, but exact label wording varies by product, jurisdiction, and regulatory history.^248,398^ Because randomized and systematic-review evidence remains mixed in routine fracture populations, these modalities are best framed as selective adjuncts rather than universally applicable standards.^247,399^

The broader therapeutic landscape is best understood by separating products according to intended regulatory or clinical use: therapies with explicit fracture-healing or nonunion-related indications; products marketed for void filling, structural support, graft extension, or fusion but sometimes used to support union; products intended primarily for infected-defect or osteomyelitis pathways; and therapies authorized outside trauma or nonunion care and therefore used off-label. Many widely discussed orthobiologics fall into these adjacent or off-label categories. BMAC and PRP are commonly used in fracture and nonunion care, but the regulated element is often the preparation system or procedural framework rather than a fracture-healing-labeled biologic product.^367,400^ Similarly, DBM, cellular bone matrices, and synthetic substitutes are generally positioned as graft substitutes, graft extenders, void fillers, structural supports, or fusion adjuncts rather than as dedicated union-promoting therapies.^367,399,400^ Their use to enhance fracture or nonunion healing is therefore often indication-specific and may be off-label in intent.

A subset of substitutes instead addresses infection-related constraints. Antibiotic-loaded calcium sulfate, calcium phosphate, hydroxyapatite, or composite bone substitutes, including gentamicin-eluting calcium sulfate/hydroxyapatite formulations such as CERAMENT G, illustrate this infection-pathway category. These products support local antibiotic delivery, dead-space or void management, and reconstruction in infected bone defects, osteomyelitis, open-fracture-related defects, or fracture-related infection pathways, rather than directly accelerating uncomplicated aseptic fracture repair.^268,269,405,406^ Their intended clinical role is therefore best understood as infection control and local defect management that may enable subsequent union, rather than direct biological stimulation of fracture healing.^407^

Other translated biologics and osteoanabolic approaches, including PDGF-BB/β-TCP combinations, P-15 peptide formulations, and systemic anabolic agents such as teriparatide, have been clinically translated mainly in adjacent bone-regeneration, fusion, spine, or osteoporosis contexts rather than as established long-bone fracture-healing therapies. Their use in fracture or nonunion care remains exploratory, adjunctive, or off-label depending on the setting, with limited or inconsistent comparative evidence for long-bone fracture and nonunion indications.^300,408–410^

Table 3 groups modalities according to these intended regulatory-use categories. Overall, despite an expanding toolbox of biologics, substitutes, procedure-based orthobiologics, and devices, only a small subset currently combines explicit fracture-healing or nonunion labeling with robust comparative evidence for fracture repair.

**Table 3:** Regulatory status and label positioning of fracture-related therapies and adjuncts in the EU

Label intent group	Therapy category (examples)	Regulatory status (EU)	Labeled/marketed use (summary)
Standard surgical grafting (no marketed product claim)	Autologous intraoperative bone graft	Not a marketed product; conventional intraoperative autologous grafting within the same surgical procedure is generally governed by surgical practice and hospital/national frameworks rather than medicinal-product marketing-authorization pathways.	Practice-based adjunctive grafting; no product label.
	Allogeneic bone graft	Supplied via tissue establishments under the EU tissues-and-cells framework (Directive 2004/23/EC); transitioning to the SoHO Regulation (EU) 2024/1938.	Marketed for osteoconductive grafting, structural support, void filling, or reconstruction; explicit fracture-healing claims are product-specific.
Selected fracture-healing or nonunion-labeled adjuncts	rhBMP-2/dibotermin alfa, e.g., InductOs	Centrally authorized medicinal product, EMA.	Authorized for selected lower-back spine fusion surgery and tibial-fracture surgery as an adjunct to standard care; use should follow the current EMA product information.
	PEMF bone stimulation	Representative products are CE-marked active therapeutic devices under the EU medical-device framework; classification and labeling are product-specific.	Labeled use is product-specific; may include nonunion and/or delayed-union indications where stated in the EU product documentation.
	LIPUS bone stimulation	Representative products are CE-marked active therapeutic devices under the EU medical-device framework; classification and labeling are product-specific.	Labeled use is product-specific; may include nonunion and/or selected fracture indications where stated in the EU product documentation.
Bone void fillers/graft substitutes	Demineralized bone matrix (DBM)	Product- and jurisdiction-dependent: may follow the tissues-and-cells framework, the medical-device pathway under the EU Medical Device Regulation (MDR), or another classification depending on processing, composition, intended use, and national implementation; generally no fracture-healing–specific authorization.	Marketed for void filling, graft extension, or graft substitution; fracture-healing claims are product-specific.
	Cellular bone matrices (CBM)	Product- and jurisdiction-dependent: may fall under the tissues-and-cells framework or, if substantially manipulated or intended to perform a different essential function in the recipient than in the donor, under Regulation (EC) No 1394/2007 as an Advanced Therapy Medicinal Product (ATMP); classification is conditional and product-specific.	Marketed as graft substitutes or graft extenders; long-bone fracture-healing claims are product-specific.
	Synthetic bone substitutes (CP ceramics/cements, calcium sulfate, bioactive glass)	CE-marked implantable medical devices under Regulation (EU) 2017/745, the EU Medical Device Regulation (MDR).	Labeled for bone void filling, augmentation, or structural support according to the specific CE-marked product documentation.
Antibacterial bone void fillers/local antibiotic carriers	Antibiotic-eluting carriers (e.g., gentamicin–calcium sulfate/phosphate composites)	Typically regulated as medical devices incorporating a local antibiotic component where the medicinal action is ancillary; classification is product-specific.	Labeled for local antibiotic delivery and/or dead-space or void management according to the specific product documentation (e.g., gentamicin-loaded calcium sulfate/hydroxyapatite composites used in osteomyelitis or infected-defect pathways where specified in product documentation).
Common off-label adjuncts for fracture union support	Platelet concentrates (PRP/PRF)	Autologous preparation; preparation systems may be CE-marked devices under Regulation (EU) 2017/745; downstream use governed by national blood/SoHO frameworks; no fracture-healing label.	Preparation-system claims only; no class-level EU fracture-healing label.
	Bone marrow aspirate concentrate (BMAC)	Autologous point-of-care preparation; processing systems may be CE-marked; classification depends on manipulation, intended function and same-procedure use and may fall under national procedural or tissue/cell frameworks or, where ATMP criteria are met, Regulation (EC) No 1394/2007.	Preparation-system claims only; therapeutic fracture-healing claims depend on product classification and documentation.
	Systemic osteoanabolic agents (e.g., teriparatide)	Authorized medicinal products for osteoporosis and fracture-risk reduction; not authorized for fracture-repair acceleration.	Authorized for osteoporosis-related indications and fracture-risk reduction; not authorized for fracture-repair acceleration.
	Other translated biologics (e.g., rhPDGF-BB/β-TCP or P-15 formulations; product examples are jurisdiction-specific)	Product- and jurisdiction-dependent; products may be authorized, CE-marked, cleared, or otherwise marketed for non-fracture indications such as spinal fusion, foot/ankle fusion, or other adjacent bone-regeneration indications, but there is no class-level EU long-bone fracture-healing authorization.	Labeled or marketed for product-specific non-fracture indications such as spinal fusion, foot/ankle fusion, or other adjacent bone-regeneration indications; application in long-bone fracture care or acute trauma should be treated as off-label unless supported by the specific product documentation.

The table summarizes EU regulatory pathways and labeled or marketed uses discussed in the main text, grouping modalities by intended label position: explicit fracture-healing or nonunion indications, void filling or fusion positioning, infection-pathway uses, and commonly used off-label adjuncts. Regulatory classification and labeled claims vary by product, processing, intended use, and Member State implementation. CE marking, tissue-establishment supply, hospital governance, ATMP classification, and medicinal-product marketing authorization are not interchangeable regulatory concepts; the table should therefore be read as a label-positioning summary rather than as a comparison of equivalent approval pathways

*ATMP* advanced therapy medicinal product, *BMAC* bone marrow aspirate concentrate, *CBM* cellular bone matrix, *CE* Conformité Européenne, *DBM* demineralized bone matrix, *EMA* European Medicines Agency, *MDR* Medical Device Regulation, *LIPUS* low-intensity pulsed ultrasound, *PEMF* pulsed electromagnetic fields, *PRP* platelet-rich plasma, *SoHO* substances of human origin

#### Clinical pipeline: early- and late-phase therapies

This section focuses on novel regenerative and advanced biologic product categories, rather than standard grafting, established BMP products, biophysical stimulation devices, or widely used off-label adjuncts discussed above. As summarized in Table 4, the fracture-healing-specific regenerative pipeline remains limited, with few late-stage programs and a larger number of early-phase, feasibility, or signal-generating studies. Most identified platforms are cell-based, tissue-engineered, or scaffold-assisted constructs rather than mature products with confirmatory fracture-specific evidence.

**Table 4:** Clinical pipeline of fracture-healing–specific investigational therapies

Trial ID	Intervention	Modality/mechanism	Indication	Phase/design	Status	Sponsor/origin
NCT07112443	NVD-003	Autologous tissue-engineered osteogenic graft	Congenital pseudarthrosis of the tibia, pediatric	Phase III, randomized	Recruiting	Novadip Biosciences
NCT05693558/EudraCT 2022-001282-12	NVD-003	Autologous tissue-engineered osteogenic graft	Congenital pseudarthrosis of the tibia, pediatric	Phase I, proof-of-concept	Unkown	Novadip Biosciences
EudraCT 2018-000299-13/NCT06335394	NVD-003	Autologous tissue-engineered osteogenic graft	Recaltritant lower-limb nonunion	Phase I/II, single-arm	Active	Novadip Biosciences
NCT05987033	NVDX3	Allogeneic cell-based osteogenic construct	Distal radius fracture	Phase I/II, single-arm proof-of-concept	Completed	Novadip Biosciences
NCT02020590	ALLOB	Allogeneic osteoblastic cell therapy	Delayed union	Phase I/IIa, open-label pilot	Completed	BioSenic (formerly Bone Therapeutics)
NCT04432389	ALLOB	Allogeneic osteoblastic cell therapy	Acute tibial fracture	Phase IIb	Unknown	BioSenic (formerly Bone Therapeutics)
NCT03024008	BonoFill-II	Autologous adipose-derived osteogenic cell and scaffold construct	Large bone defects/nonunion	Phase I/II, single-arm	Recruiting	Bonus BioGroup
NCT01842477	OrthoCT1	Autologous expanded MSCs and ceramic scaffold	Delayed union/nonunion	Phase II	Completed	INSERM consortium

Table 4 summarizes registry-listed clinical studies of defined fracture-healing product platforms. Trial phase/design, indication, sponsor/origin, and recruitment status are taken from the registry records identified in the Trial ID column. Registry information was checked in August 2026 and may evolve over time. “Unknown” indicates that an active study record has not been updated within the required registry period and does not mean that the study was terminated

*EudraCT* European Union Drug Regulating Authorities Clinical Trials Database, *MSC* mesenchymal stromal cell

NVD-003, an autologous tissue-engineered osteogenic graft, is among the more clinically advanced examples. Its development spans difficult bone-healing indications, including congenital pseudarthrosis of the tibia and severe lower-limb nonunion, but these indications should be interpreted separately rather than as a single unified clinical program. The most advanced current study is the pediatric congenital pseudarthrosis trial, in which NVD-003 is being evaluated in a randomized Phase III comparison with iliac crest bone graft (NCT07112443; Table 4), whereas earlier adult lower-limb nonunion studies are better viewed as early-phase or long-term follow-up experience rather than evidence of broad clinical applicability.

A multicenter, single-arm Phase III study from Japan evaluating autologous peripheral blood CD34⁺ cell transplantation for femoral and tibial nonunion provides an additional academic late-stage example. Despite encouraging healing signals, its single-arm design, small cohort, and reliance on historical controls mean that it should be interpreted cautiously rather than as confirmatory comparative evidence.^323^

Other programs listed in Table 4 remain mainly early phase and include allogeneic osteoblastic cell therapy, autologous cell/scaffold constructs, and expanded MSC-based scaffold approaches evaluated in delayed union, nonunion, acute fracture, or bone-defect settings. These studies demonstrate clinical deliverability and may generate early safety or healing signals, but most remain focused on feasibility, safety, or preliminary efficacy rather than definitive comparative evidence.

EV-based and gene-directed strategies remain largely preclinical or very early translational in fracture repair and are not yet part of the established clinical fracture-healing pipeline.^354,357^ Overall, early clinical activity is broader than late-stage progress, and progression toward confirmatory evaluation remains limited.^379,411^

### Manufacturing quality and scalability

Manufacturing design is a major determinant of whether fracture-healing therapies can move beyond small trials into routine practice. Commercially deployed products must be produced within appropriate validated quality systems, with requirements depending on product class and regulatory route.^412,413^ For MSC-based and other cell-based advanced therapies, this also requires clinically meaningful potency assays, reproducible release criteria, and tight control of batch-to-batch variability linked to in vivo function.^414,415^

Scaling from pilot production to multicenter or commercial use is particularly challenging for cell-based products and complex combination therapies. Centralized GMP manufacture supports process control but increases logistical complexity and may be poorly suited to time-sensitive fracture and trauma workflows, whereas point-of-care processing is surgically flexible but harder to validate, standardize, and compare across sites. Decentralized or hybrid manufacturing models are therefore being explored as pragmatic compromises.^416^

For personalized or small-batch ATMPs, closed and automated systems and in-process analytics are increasingly being used or developed to limit variability and support data integrity.^416–418^ However, these approaches add technical complexity, training requirements, and implementation costs, leaving many cell-based and combination products labor-intensive, difficult to standardize, and vulnerable to variability as scale increases.^419^

In fracture healing, this challenge is particularly acute because many indications are common, surgically integrated, and time-sensitive. Manufacturing should therefore be treated as part of the clinical-development strategy rather than as a downstream operational issue.

### Safety and Ethics

Safety oversight for fracture-healing therapies follows the usual pharmacovigilance and device-surveillance frameworks, but several issues are especially important in this field.^367,395^ For medicinal products, biologics, and ATMPs, regulatory analyses emphasize adverse-event surveillance, risk minimization, long-term follow-up, registry-based monitoring, traceability, and, where relevant, biodistribution or integration-site assessment.^367,395,420^ Higher-risk devices and combination products, such as BMP-scaffold systems or active stimulators, also require attention to postmarket clinical performance and delayed or uncommon complications, including ectopic bone formation, inflammatory reactions, implant failure, or persistent nonunion.^375,376^

In fracture healing, safety assessment is complicated because adverse outcomes are often difficult to attribute to a single cause. Delayed union, nonunion, implant failure, infection, and soft-tissue complications may reflect the product, procedure, mechanical environment, host factors, or their interaction. Long-term follow-up and clear event adjudication are therefore particularly important, especially for advanced biologics and combination products.^381,382^

Ethical considerations are also prominent because fracture-healing studies often involve vulnerable populations, including children with open physes, frail older adults, and multimorbid patients with fragility fractures.^421^ Informed consent, realistic risk-benefit assessment, and fair patient selection therefore require particular care. As digital monitoring, wearables, and AI-supported decision tools become more common in musculoskeletal research and care, oversight should also address evidence generation, data governance, clinical validation, and accountability for technology-assisted decisions.^422^

Safety and ethics in fracture healing therefore extend beyond product toxicity alone. They also include long-term traceability, attribution of delayed complications, appropriate inclusion of vulnerable patients, and accountability for technology-assisted clinical decisions.

### Reimbursement and affordability

Successful translation of fracture-healing technologies depends not only on regulatory approval, but also on reimbursement, cost-effectiveness, and value demonstration.^423–426^ EMA or FDA authorization does not guarantee payer coverage, and for many regenerative products this disconnect can be a major reason why biologically active therapies fail to reach routine care. Early identification of the likely payer and reimbursement pathway is therefore essential, particularly for advanced products with high development, manufacturing, infrastructure, and labor costs.^419,425,426^

HTA is the main framework through which payers judge whether a new intervention offers sufficient value relative to existing care.^397,427^ In the EU, Regulation (2021/2282), which applies from January 12, 2025, introduced EU-level joint clinical assessments for selected product groups, including ATMPs, with broader implementation planned over time.^428^ Fracture-healing developers must therefore generate evidence that satisfies both regulatory requirements and payer expectations around clinical benefit, functional recovery, durability, and cost offsets.

In fracture healing, economic value is driven less by mortality than by avoidance of reoperation, shortening of disability, and earlier restoration of function. Cost-effectiveness analyses should therefore capture direct medical costs as well as downstream effects such as return to work, productivity loss, and prolonged rehabilitation. Tibial-fracture modeling is particularly informative: nonunion can substantially increase two-year costs, although the magnitude varies by fracture type, infection status, healthcare system, costing perspective, and whether indirect productivity losses are included.^4,429^ Consistent with this, one UK HTA model of BMP-2 for open tibial fractures estimated an incremental cost-effectiveness ratio of £32,603 per quality-adjusted life year (QALY) gained, illustrating how improvements in union or healing time may materially affect cost-effectiveness if they reduce reoperation and sick leave.^430^

Reimbursement barriers differ by product class. Conventional drugs often fit HTA frameworks relatively well because manufacturing is standardized, risk profiles are familiar, and costs may decrease with scale. ATMPs are more difficult because production is often individualized or technically complex, infrastructure requirements are high, and long-term effectiveness may remain uncertain at assessment.^426,431^ This creates a difficult payer proposition, especially for one-time high-cost interventions whose value cannot easily be recovered if outcomes fall short.^432^ Minimally manipulated autologous or allogeneic products may occupy a further gray zone, with variable classification, heterogeneous coverage routes, and limited long-term evidence.^367^

Medical devices and synthetic substitutes face different pressures. Their regulatory pathway is often clearer and manufacturing more predictable, but payers still require evidence that premium pricing is justified by superior outcomes or measurable cost offsets. This is particularly relevant for products positioned against autograft or standard reconstructive materials, antibiotic-eluting substitutes intended to reduce recurrent infection or enable single-stage treatment, and drug-device combinations that increase total intervention costs.^397,427^

Coverage processes and evidence expectations vary across payers, making early payer engagement essential. Experience with early ATMPs illustrates this clearly. Glybera, the first gene therapy approved in Europe, is often cited as an example of how regulatory approval, high upfront cost, and reimbursement uncertainty can limit clinical uptake.^433,434^ Although Glybera lies outside fracture healing, the lesson is directly relevant. Early health-economic modeling, payer consultation, and incorporation of payer-relevant outcomes into development programs can reduce this risk.^435^ In fracture healing, particularly relevant outcomes include validated patient-reported outcome measures (PROMs), reoperation, return to weight-bearing, return to work, and plans for post-launch real-world evidence generation.^436^

Access is also shaped by geography and health-system capacity. Advanced biologics and regenerative therapies are more likely to reach patients in systems with established reimbursement mechanisms, specialist centers, trained personnel, and manufacturing or logistics infrastructure.^437^ In low- and middle-income settings, access barriers are amplified by high treatment costs, limited infrastructure, and the fact that clinical development has largely occurred in high-income countries.^438^ These disparities raise equity concerns and highlight the need to consider affordability, implementation feasibility, and health-system capacity when evaluating regenerative therapies for fracture care.

A range of reimbursement models has been proposed to manage high upfront costs and uncertainty around long-term benefit, including outcome-based managed entry agreements, risk-sharing arrangements, and staggered or installment-style payment systems.^432,437^ However, these models are administratively demanding and require agreement on financial terms, governance, outcome-data collection, registry infrastructure, and mechanisms linking payment to real-world performance.^437^ In fracture healing, applying such models would require consensus on payer-relevant outcomes such as union, reoperation, return to weight-bearing, functional recovery, and validated patient-reported outcome measures.

Reimbursement and affordability are therefore not secondary implementation issues, but core determinants of whether fracture-healing technologies achieve real-world adoption. Clinical benefit must be supported by credible economic value, payer-relevant outcomes, and a practical route to coverage.

## Challenges and future perspectives

Fracture repair is increasingly understood as a systems-level regenerative process rather than a purely local osteogenic process, shaped by coordinated immune, neural, mechanical, and systemic regulatory networks. In this view, impaired healing reflects failure of integrated biological and biomechanical regulation rather than disruption of any single pathway. Future fracture-healing strategies will therefore likely depend less on isolated therapies than on patient-specific combinations of biological augmentation, mechanical optimization, and monitoring tailored to individual healing trajectories.

### Scientific and clinical gaps

#### Complexity and inter-patient variability

Fracture healing is a complex, multistage process governed by tightly coordinated cellular and molecular events.^8^ Age, sex, genetics, metabolic bone disease (e.g., osteoporosis, diabetes), medications, lifestyle, and comorbidities all influence healing outcomes, so patients with comparable fractures may follow markedly different trajectories, from rapid union to delayed healing or nonunion despite optimal care.^151^ A central challenge is that impaired fracture healing is still often approached as a relatively uniform clinical entity, even though the dominant biological bottleneck may differ substantially between patients, for example insufficient vascularization, impaired osteogenesis, or dysregulated inflammation. Patient-specific healing trajectories remain difficult to predict in routine practice. Emerging imaging-based and biomarker-driven approaches may support more granular assessment of nonunion risk, but these approaches are not yet validated for routine clinical use.^152,439^ Improving outcomes will therefore likely require better integration of biological, genetic, mechanical, and environmental determinants to define patient-specific failure modes and guide tailored intervention.

#### Lack of predictive preclinical models

Translation of basic fracture-healing research into effective clinical therapies remains limited by differences between current preclinical models and clinical fracture populations. Many animal and In vitro systems rely on young, healthy, genetically uniform subjects, whereas real-world patients are often older, multimorbid, and metabolically heterogeneous. Experimental fixation strategies and loading conditions may also fail to reflect clinical biomechanics.^176,440^ This mismatch may reduce translational relevance and likely contributes to the high attrition of fracture-healing therapies in clinical development. Emerging human-based platforms, including fracture hematoma models, 3D fracture-gap constructs, microfluidic co-culture systems, and mechanobiology-informed in silico simulations, may improve translational relevance, but they remain heterogeneous and insufficiently standardized for comparative testing.^225^ Future progress will require stratified experimental systems that incorporate clinically relevant modifiers such as age, diabetes, smoking, inflammatory disease, and altered mechanical loading. Therapies will be more predictive only if tested under realistic failure conditions.^440,441^

### Precision medicine and stratified approaches

#### Biomarker-guided personalization

Recognition of fracture-healing heterogeneity has intensified efforts to identify biomarkers that predict individual regenerative capacity and support personalized therapeutic strategies. Candidate biomarkers span molecular, cellular, imaging, and genetic domains, but differ substantially in biological specificity and translational maturity. Circulating protein markers, including TGF-β1, and inflammatory mediators such as IL-6, TNF-α, and IL-1β have been investigated in relation to delayed healing and nonunion risk, reflecting the importance of inflammatory resolution and osteogenic signaling.^442,443^ Cellular biomarkers, including altered B-cell activity and expansion of myeloid-derived suppressor cells, further implicate immune dysregulation in impaired repair.^443,444^ Circulating miRNAs are also promising because of their stability in circulation and their regulatory roles in osteogenesis, angiogenesis, and inflammation, although most remain at the discovery or early validation stage.^445^ Imaging-derived biomarkers, including quantitative computed tomography (CT)-based callus density, callus volume, microarchitecture, and radiographic or CT-based healing scores, are particularly attractive because they provide spatially resolved and longitudinal information on healing progression.^446–449^ By contrast, genetic and epigenetic markers offer mechanistic insight but remain largely exploratory. Despite extensive research, no single biomarker or biomarker panel has entered routine clinical use, largely because of variability in study design, limited cohort sizes, and lack of prospective validation.^443,450^ These limitations have encouraged interest in multimodal “healing profiles” that integrate molecular, cellular, imaging, and clinical data into composite risk models.^450^ Overall, imaging-integrated and multimodal strategies appear most suitable for further validation, whereas serum cytokines remain limited by low specificity, circulating miRNAs are still early-stage, and genetic or epigenetic markers remain exploratory. The key next step is not merely biomarker discovery, but robust prospective validation and demonstration that biomarker-guided decision-making improves outcomes compared with standard care.

#### AI and digital monitoring

Digital technologies and AI may support personalized fracture care by enabling more continuous and quantitative assessment of healing. Conventional radiographs and CT scans provide episodic information and may not reliably capture early biological or mechanical deviations. Smart sensing technologies, including implantable load sensors, accelerometers, and ultrasonic monitoring systems, can generate longitudinal data on callus stiffness, load sharing, and implant stability. By tracking changes in mechanical behavior over time, these approaches may identify deviations before radiographic delayed union or union becomes apparent.^451,452^ Machine-learning approaches are being explored to integrate imaging features, sensor-derived data, and clinical variables into predictive models of delayed healing and nonunion. Current evidence should be interpreted as proof-of-concept and early translational rather than as externally consolidated clinical validation. Adaptive algorithms may eventually update risk estimates as new data become available, but this remains feasibility-level and requires validation.^392^ Major barriers remain, including the need for external validation, interoperability with clinical workflows, standardized data acquisition, bias mitigation, and regulatory approval. In the near term, the greatest value of these technologies may lie in risk stratification, remote monitoring, and objective follow-up of high-risk fractures rather than fully automated treatment selection.

#### Demographic-tailored strategies

Therapeutic stratification should account for age, skeletal maturity, osteoporosis status, and comorbidity burden. Older adults often exhibit impaired osteogenesis, chronic low-grade inflammation, and reduced mechanosensitivity,^156,158^ whereas children and young adults generally retain greater endogenous regenerative capacity.^8^ Postmenopausal women represent a subgroup in whom hormonal deficiency and bone loss may alter repair biology.^166^ At present, however, evidence remains insufficient to recommend distinct fracture-healing therapeutic algorithms solely on the basis of demographic category. These variables are better incorporated into broader biologic risk stratification than used as stand-alone treatment determinants. Integrating demographic features with biomarkers, imaging findings, comorbidity profiles, and mechanical-risk assessment may help identify biologically vulnerable patients, but requires prospective validation before guiding routine fracture-healing interventions.

### Emerging directions

Recent fracture-healing research increasingly recognizes that impaired healing can reflect systems-level failure involving immune dysregulation, neural signaling, and systemic host–environment interactions rather than a purely local defect of osteogenesis. Emerging therapeutic strategies therefore increasingly target these broader regulatory networks. Representative examples are highlighted below together with their main translational constraints.

#### Immuno-regeneration

Targeted modulation of the immune–skeletal interface is an important emerging direction in fracture repair. Effective healing requires a tightly regulated inflammatory response: early immune activation supports progenitor recruitment and repair initiation, whereas prolonged or dysregulated inflammation disrupts callus formation.^453^ Preclinical studies show that manipulating immune-cell phenotype can enhance bone repair, including through macrophage polarization, macrophage-derived EVs, and immunomodulatory biomaterials.^454–458^ For example, local transplantation of CD14⁺ macrophage precursors into the osteotomy gap of aged rats partially rescued impaired bone regeneration after 6 weeks, increasing new bone deposition, reducing fibrosis, and improving callus vascularization; these findings support a contributory role for impaired M2-associated macrophage function in age-related healing deficits.^458^ M2 macrophage-derived exosomes have likewise been shown to reprogram the osteoimmune microenvironment by reducing M1 macrophage proportions and promoting M1-to-M2 conversion through PI3K/AKT signaling, thereby accelerating fracture healing in diabetic models.^459^ In addition, aptamer-functionalized M2 macrophage exosomes targeted to MSCs enhanced fracture-site accumulation and promoted healing in murine femoral fracture models.^460^ Together, these findings provide preclinical support for immuno-regenerative fracture therapies. However, translation remains constrained by manufacturing, scalability, delivery, and timing. Cell- and exosome-based products are difficult to standardize clinically, and effective use will likely require temporal control that preserves the balance between inflammatory activation and resolution. Clinical implementation therefore remains limited despite promising experimental efficacy.

#### Bone-nerve interaction

Bone–nerve interaction is increasingly recognized as an important regulator of fracture healing. Preclinical and review evidence indicates that the peripheral and autonomic nervous systems influence fracture repair through neuropeptide/neurotrophin signaling, effects on angiogenesis, and crosstalk with immune and skeletal cells. Key mediators include CGRP, SP, neuropeptide Y, NGF, and brain-derived neurotrophic factor (BDNF), which have been linked to inflammation, osteogenesis, endochondral ossification, remodeling, and fracture pain.^461,462^ β-NGF has therefore been explored as a local regenerative cue, including sustained delivery approaches: β-NGF-loaded PEGDMA microrods improved callus bone fraction, trabecular connectivity, and bone mineral density compared with soluble β-NGF control, while reducing cartilage fraction.^463^ However, translation remains limited by safety and delivery concerns. NGF is strongly implicated in pain signaling: NGF receptors are expressed by bone nociceptors, and direct tibial NGF injection in animal models rapidly activates and sensitizes these nociceptors.^464^ Clinical fracture-healing applications would therefore require tightly controlled local or sustained delivery, dose optimization, and strategies to limit nociceptive adverse effects. At present, the strongest support is preclinical, and clinical fracture-healing data, optimal timing, and dosing remain insufficiently established.

#### Microbiome-based therapies

Emerging preclinical evidence suggests that the gut microbiome may influence bone repair by shaping systemic immune responses relevant to healing. However, the current evidence base remains early, largely preclinical, and heterogeneous, and fracture-specific mechanistic data are still limited. In mice, antibiotic-induced microbiome depletion or dysbiosis reduced the expansion of IL-17–expressing Th17 cells and impaired callus formation.^206^ Microbiota-derived metabolites may also influence bone anabolism more broadly; in mice, butyrate produced by intestinal microbiota was required for PTH-induced bone formation through T-cell-mediated signaling.^465^ Microbiota-targeted interventions therefore remain exploratory in the context of fracture repair. Major challenges include inconsistent human data, limited standardization, and unclear regulatory pathways. Their future relevance will depend on demonstrating reproducible clinical effects and identifying the patient groups in whom systemic immune modulation meaningfully improves repair.

## Conclusion and outlook

Fracture healing is now understood as a coordinated, multistage regenerative process shaped by inflammatory resolution, progenitor recruitment, angiogenesis, lineage specification, mechanoregulation, matrix remodeling, and restoration of skeletal architecture. The classical sequence of hematoma formation, inflammation, soft and hard callus formation, and remodeling remains a useful framework, but current evidence shows that successful repair depends on broader interactions among immune, vascular, skeletal, neural, stromal, and mechanical systems. These interactions are regulated by temporally and spatially controlled signaling networks, including cytokine-mediated inflammation, hypoxia- and VEGF-driven angiogenesis, TGF-β/BMP and Wnt-mediated lineage specification, Hedgehog and Notch signaling, mechanotransduction, and osteoclast–osteoblast coupling. This integrated view helps explain why fracture repair can fail in different ways, including delayed union, nonunion, and mechanically inferior repair.

A major implication of this framework is that impaired healing should not be viewed as a single biological defect. Failure can result from persistent inflammation, impaired vascularization, insufficient progenitor recruitment, defective osteogenic or chondrogenic differentiation, infection, mechanical instability, systemic disease, or abnormal matrix remodeling. These factors often overlap, particularly in patients with aging, osteoporosis, diabetes, smoking, chronic kidney disease, obesity, vascular compromise, or hormonal deficiency. As a result, therapies that appear effective in controlled experimental systems may not translate directly to heterogeneous clinical populations. This remains a central challenge in the field: fracture repair is governed by highly context-dependent pathways, where the same signal may support regeneration at one stage but become harmful if excessive, prolonged, or delivered in the wrong mechanical or biological environment.

Clinical fracture care therefore continues to rely on established principles: stable fixation, restoration of alignment and structural integrity, infection control, appropriate rehabilitation, and graft- or substitute-based augmentation when indicated. At the same time, therapeutic development has expanded beyond conventional reconstruction. Growth factors, osteoanabolic agents, bone substitutes, biophysical stimulation, cell-based therapies, gene- and RNA-based approaches, extracellular vesicles, immunomodulatory biomaterials, neuroregenerative cues, microbiome-related strategies, and tissue-engineered constructs all aim to target specific biological constraints in repair. However, many remain preclinical or early clinical, with incomplete and indication-specific evidence for added benefit over established care. Clinical implementation is further limited by uncertainty around patient selection, timing, dosing, delivery, durability, manufacturing reproducibility, safety, regulation, cost-effectiveness, and reimbursement.

Future progress will likely depend on integrating biological insight with sound orthopedic principles rather than replacing foundational care. More precise patient stratification, validated biomarkers, quantitative imaging, objective monitoring technologies, and clinically relevant preclinical models may help identify which patients are most likely to benefit from specific interventions. Clinical trials will also need clearer alignment between therapeutic mechanism, fracture indication, patient risk profile, delivery strategy, and outcome measures. The most realistic near-term advances may come from combining optimized fixation and rehabilitation with targeted biological augmentation in selected high-risk patients. In the longer term, multimodal risk profiling and mechanism-based trial design may help make fracture treatment more predictable, personalized, and effective for patients at greatest risk of delayed union, nonunion, or functionally inadequate repair.
